# The role of self-confidence in teamwork: experimental evidence

**DOI:** 10.1007/s10683-024-09829-x

**Published:** 2024-05-18

**Authors:** Adrian Bruhin, Fidel Petros, Luís Santos-Pinto

**Affiliations:** 1 Faculty of Business and Economics (HEC Lausanne), University of Lausanne, Lausanne, Switzerland; 2 WZB Berlin and TU Berlin, Berlin, Germany; 3 Faculty of Business and Economics (HEC Lausanne), University of Lausanne, Lausanne, Switzerland

**Keywords:** Teamwork, Self-confidence, Effort, Free riding, C71, C92, D91, D83

## Abstract

Teamwork has become increasingly important in modern organizations and the labor market. Yet, little is known about the role of self-confidence in teamwork. In this paper, we present evidence from a laboratory experiment using a team effort task. Effort and ability are complements and there are synergies between teammates’ efforts. We exogenously manipulate subjects’ self-confidence about their ability using easy and hard general knowledge quizzes. We find that overconfidence leads to more effort, less free riding, and higher team revenue. This finding is primarily due to a direct effect of overconfidence on own effort provision, while there is no evidence that subjects strategically respond to the teammate’s overconfidence.

**Supplementary Information:**

The online version contains supplementary material available at 10.1007/s10683-024-09829-x.

## Introduction

This paper studies the role of self-confidence in teamwork using a laboratory experiment. We address the following questions. First, to what extent does overconfidence raise effort, mitigate free riding, and increase revenue in teams? Second, what potential channels are at play? Third, can overconfidence increase individual and team payoffs?

Existing studies show that overconfidence matters for labor markets (Hoffman et al., 2020; Santos-Pinto et al., 2020; Dargnies et al., 2019). In particular, it changes firms’ design of labor contracts (Sautmann, 2013; de la Rosa, 2011; Santos-Pinto, 2008), workers’ choice of compensation schemes (Dohmen & Falk, 2011; Niederle et al., 2007), as well as entry and behavior in tournaments (Huffman et al., 2022; Möbius et al., 2022; Dohmen et al., 2011; Santos-Pinto, 2010; Niederle et al., 2007).

However, the effect of overconfidence on teamwork has received less attention. This lack of attention is surprising given the growing importance of teamwork. According to Lazear and Shaw (2007), teamwork in US firms has increased substantially since the 1980s. For example, from 1987 to 1996, the share of large firms with more than a fifth of their workers in problem-solving teams rose from 37% to 66%. The percentage of large firms with workers in self-managed teams rose from 27% to 78%. The importance of teamwork has also been growing in the mutual fund industry, where 76% of US mutual funds are currently managed by a team (Rodríguez-Revilla & García-Gallego, 2023). Another example is the trend of increasing co-authorship in economics, where the share of co-authored papers increased from 50% in 1996 to 75% in 2014 (Jones, 2021; Kuld & O’Hagen, 2020; Barnett, Ault, and Kaserman, 1988).

An exception is Gervais and Goldstein (2007) who take the effects of overconfidence on teamwork explicitly into account. They show theoretically that overconfidence may raise effort and mitigate free riding in teams. In their model, team revenue increases in players’ abilities and efforts. That is, a player’s marginal productivity of effort increases in own ability (effort and ability are complements) and the effort of others (efforts are strategic complements). There are two channels by which overconfidence raises efforts and reduces free riding. First, as effort and ability are complements, the overconfident player exerts more effort himself. Second, as efforts are strategic complements, the teammate anticipates the overconfident player’s higher effort and increases her effort in turn. Hence, the presence of an overconfident player, i.e., someone overestimating his marginal productivity, leads to less free riding and, thus, may make all players better off, including the biased player himself.

In this study, we experimentally test the predictions of this model and identify the importance of the two channels. The experiment closely mirrors the model’s features. It randomly assigns subjects into pairs and exposes them to a team task where they choose their individual efforts simultaneously.^1^ In this task, team revenue reflects the complementarity between individual effort and ability as well as the strategic complementarity between the two subjects’ efforts. A subject’s ability in the team effort task corresponds to his rank in a general knowledge quiz among a group of twelve randomly matched subjects. We exogenously manipulate beliefs about ability using a between-subjects design, which exposes subjects either to an easy or a hard quiz. This manipulation of beliefs exploits that subjects overplace themselves in easy tasks and underplace themselves in hard tasks (Moore & Healy, 2008; Moore & Kim, 2003; Krueger & Mueller, 2002; Kruger, 1999; Kruger et al., 1999).^2^

To measure a subject’s self-confidence bias, we compare his true rank in the general knowledge quiz to his belief. We elicit the belief before the team effort task to mitigate strategic incentives to misreport beliefs and incentivize them using a binarized scoring rule (Hossain & Okui, 2013). If the subject’s belief exceeds his true ability, he is overconfident; if it falls short of his true ability, he is underconfident.

The effort task is a team version of the ball catching task by Gächter et al. (2016). Each subject belongs to a team of two and has to move a tray to catch balls randomly falling from the top of the screen.^3^ Effort corresponds to the number of clicks to move the tray and has a constant marginal cost. The marginal contribution of a catch to team revenue increases in the subject’s ability and the teammate’s number of catches. This ensures that effort and ability are complements, and subjects’ efforts are strategic complements. Team revenue is split equally between the teammates.

Each subject performs the effort task over eight periods with the same teammate. The subject does not know his own ability. However, the subject observes his teammate’s ability and belief about her ability, allowing him to infer the teammate’s self-confidence bias. This is crucial for distinguishing the two channels through which overconfidence can raise effort and mitigate free riding. While the first channel – relying on effort and ability being complements – directly increases an overconfident subject’s effort, the second channel – relying on the strategic complementarity of efforts – requires that each subject is aware of his teammate’s self-confidence bias.^4^

At the end of each period, the subject observes his own number of clicks and catches as well as his payoff. However, he does not observe the teammate’s catches, clicks, and payoff. This ensures that the disclosure of payoff information does not allow the subject to calculate his true ability.

The results show that the belief manipulation worked. Beliefs reveal that subjects exposed to the easy quiz are overconfident and, on average, overestimate their rank by 1.2 places, while those exposed to the hard quiz are underconfident and, on average, underestimate their rank by 0.483 places.

The results also confirm the theory’s main prediction – i.e., overconfidence increases effort, thus reducing free riding. Subjects exposed to the easy quiz provided 21.1% more effort than those exposed to the hard quiz. We also show that an exogenous shift in beliefs causes the treatment differences in effort provision,^5^ rules out that mood effects are driving this result.

Furthermore, we uncover that the increase in effort and reduction in free riding is primarily due to the first channel, as a subject’s self-confidence bias has a positive effect on own effort. However, there is no evidence for the second channel, as own effort does not react to the teammate’s self-confidence bias.^6^ We also find that team revenue increases in self-confidence bias. In addition, a subject’s payoff increases in the teammate’s self-confidence bias and is inversely u-shaped in his own bias.

These results have direct implications for organizations and labor economics. While worker overconfidence can be detrimental in many settings, we confirm that it can also have positive effects in the context of teamwork. If effort and ability are complements, overconfident workers may improve team performance by reducing free riding. In addition, the absence of the second channel suggests that disclosing the degree of overconfidence among the members of a team may not be a fruitful approach to increasing team performance.

The paper contributes to two strands of literature. First, it adds to the literature on teamwork and public good provision (for a comprehensive overview, see Drouvelis, 2021). The seminal theory contribution on teamwork is by Holmström (1982). In this model, workers have an incentive to free ride whenever the efforts of other teammates are unobservable and team revenue is shared. Consequently, teamwork produces a social dilemma in which individually rational decisions lead to an inefficient outcome. However, the experimental literature on teamwork and public good provision finds that subjects do not systematically free ride. Moreover, heterogeneity in teammates’ preferences, beliefs, and demographics affect team production (Ivanonva-Stenzel & Kübler, 2011; Lavy, 2002; Knez et al., 2001; Nalbantian et al., 1997). For instance, social preferences reduce free riding in teams, as some are willing to incur costs to punish free riders (Falk et al., 2006; Kandel et al., 1992), others view free riding as a violation of social norms (Fehr & Fischbacher, 2004; Fehr & Gächter, 2000), and some are conditional cooperators (Sherstyuk et al., 2002; Fischbacher et al., 2001; Rotemberg, 1994). The paper extends this literature by analyzing the effects of overconfidence on teamwork. It is the first to show that overconfidence leads to higher efforts and less free riding and identifies the underlying channel.^7^

Second, the paper adds to the literature on overconfidence. Except for Gervais and Goldstein (2007), this literature has largely neglected the effects of overconfidence on teamwork. Seminal papers in this strand of literature have mainly focused on the impact of overconfidence on principal-agent relationships. For instance, Bénabou and Tirole (2002) and Bénabou and Tirole (2003) demonstrate that overconfidence raises the agent’s effort when effort and ability are complements. Chen and Schilberg-Hörisch (2019) show empirically that negative information on individual ability diminishes the agent’s effort provision. Our paper extends this strand of literature by providing evidence on the effects of overconfidence in a team setting, where subjects are partners without a hierarchical relationship. In that sense, the paper also links the first strand of literature on teamwork and public good provision to this second strand on overconfidence.

The paper has the following structure. Section 2 presents the theoretical model and the hypotheses we derive from it. Section 3 describes the experiment. Section 4 discusses the results on the effectiveness of the belief manipulation, the effects of self-confidence on teamwork, potential learning effects about own ability, and delayed reactions to information about the teammate. Section 5 concludes.

## Model

In this section, we present the setup of our model to study the impact of overconfidence on teamwork. Subsequently, we discuss the main hypotheses stemming from this model.

### Setup

The seminal contribution to the literature on teamwork and overconfidence is Gervais and Goldstein (2007). They consider a model of teamwork where team revenue is increasing in players’ abilities and efforts. They postulate two types of complementarities: (i) each player’s ability and effort are complements, that is, the returns to increasing effort for a high-ability player are greater than those of a low-ability player; (ii) players’ efforts are strategic complements, that is, the returns of a player’s effort are increasing in the other’s effort. Moreover, they assume a team is composed of an overconfident player and an unbiased player. The overconfident player overestimates his ability but is unaware of this bias. The unbiased player knows about the overconfident player’s bias. The overconfident player knows the unbiased player thinks that he is biased but disagrees with her. The solution concept follows the approach by Heifetz et al. (2007a, 2007b) for games with complete information and by Squintani (2006) for games with incomplete information.

We consider a modified version of this model, which retains its main features and adapts it to our experiment. A team comprises two players, *i* and *j*. Team revenue is

1 $$\begin{aligned} R = 2 w \left[ a_i \, q(e_i) + a_j \, q(e_j) + s \, q(e_i) \, q(e_j) \right] , \end{aligned}$$

where $w>0$, $a_i$ is player *i*’s ability multiplier, and $q(e_i)$ is *i*’s individual output, given by an increasing and concave function of effort. The parameter $s>0$ governs the complementarity between the two players’ efforts. It implies the two players create positive externalities on each other, as in Alchian and Demsetz (1972). Hence, *i*’s ability multiplier and effort are complements, i.e., ${\partial ^2 R}/{\partial a_i \partial e_i} >0$; and the two players’ efforts are strategic complements, ${\partial ^2 R}/{\partial e_i \partial e_j} >0$. The players are risk neutral and face linear costs of effort, $c(e_i) = c\,e_i$, with $c>0$.

Each player *i* is unaware of his ability multiplier, $a_i$, but has a belief about its value. We call this belief player *i*’s perceived ability multiplier and denote it by $\tilde{a}_i$. Thus, *i*’s self-confidence bias, $b_i$, is the difference between his perceived and true ability multipliers: $b_i=\tilde{a}_i - a_i$. When $b_i>0$, *i* is overconfident; whereas when $b_i<0$, he is underconfident. Moreover, player *i* also knows the perceived and true ability multipliers of the other player *j*. Hence, player *i* is informed about *j*’s self-confidence bias.

Team revenue is shared equally between the two players *i* and *j*, regardless of their efforts and ability multipliers. Each player *i* chooses his effort to maximize his perceived payoff

$$\begin{aligned} U_i = w \left[ \tilde{a}_i \, q(e_i) + a_j \, q(e_j) + s \, q(e_i) \, q(e_j) \right] - c\, e_i . \end{aligned}$$

For tractability and without loss of generality, we assume $q(e_i)=\sqrt{e_i}$.^8^ Under this assumption, the optimal effort levels of players *i* and *j* satisfy the following first-order conditions:

$$\begin{aligned} {\left\{ \begin{array}{ll} w \, \tilde{a}_i + s\,w \, \sqrt{e_j} = 2c \, \sqrt{e_i} \\ w \, \tilde{a}_j + s\,w \, \sqrt{e_i} = 2c \, \sqrt{e_j} \end{array}\right. } . \end{aligned}$$

Solving the above system of equations, and using $\tilde{a}_i=a_i+b_i$ as well as $\tilde{a}_j=a_j+b_j$, yields player *i*’s equilibrium effort:

2 $$\begin{aligned} e^*_i = k \, \left[ w \, (a_i+b_i) + \frac{s \, w^2}{2c} \, (a_j+b_j) \right] ^2 , \end{aligned}$$

where $k=(2c)^2/(4c^2-s^2 w^2)^2$ is a positive constant. Hence, equilibrium effort increases in the players’ ability multipliers, $a_i$ and $a_j$, and self-confidence biases, $b_i$ and $b_j$.

Our model differs from the one by Gervais and Goldstein (2007) in three relevant dimensions. First, $q(e_i)$ is concave instead of linear. Second, the cost of effort is linear instead of convex. These two dimensions map the team production to the effort task in the experiment and ensure player *i*’s second-order condition is satisfied. Third, we allow both players to be biased, whereas Gervais and Goldstein (2007) allow only one player to be biased. Even though our model differs in these three main dimensions, the qualitative predictions are identical to those derived by Gervais and Goldstein (2007), as shown in Appendix B.

### Hypotheses

We now turn to the main hypotheses. The first three hypotheses concern the effects of overconfidence on equilibrium efforts and team revenue. We obtain them directly from Eq. (2).

#### Hypothesis 1

Player *i*’s self-confidence bias has a positive effect on his equilibrium effort.

The first hypothesis follows from the assumption that a player’s ability multiplier and his effort are complements. Hence, an overconfident player overestimates the marginal productivity of his effort.

#### Hypothesis 2

Player *j*’s self-confidence bias has a positive effect on player *i*’s equilibrium effort.

The second hypothesis follows from the assumption that the players’ efforts are strategic complements. In other words, the marginal productivity of a player’s effort increases in the other’s effort. Thus, if an overconfident player exerts more effort, providing higher effort becomes more attractive to the teammate.

#### Hypothesis 3

Team revenue increases in self-confidence bias.

The third hypothesis follows directly from Hypotheses 1 and 2.

Our next three hypotheses concern the impact of overconfidence on the players’ individual payoffs and on the team payoff. We obtain them from further analysis of the model, which we detail in Appendix C.

#### Hypothesis 4

A player’s equilibrium payoff increases in the other’s self-confidence bias.

If the other player exerts more effort (due to overconfidence), team revenue is higher at no additional cost to the focal player.

#### Hypothesis 5

A player’s equilibrium payoff is inversely u-shaped in his self-confidence bias.

A player’s self-confidence bias has two effects on his equilibrium payoff: i) a direct effect resulting from the mistake in optimization and ii) a strategic effect resulting from the other’s reaction due to the strategic complementarity of the players’ efforts. The direct effect is always negative, while the sign of the strategic effect depends on whether the player is over- or underconfident. If the player is underconfident, the strategic effect is negative, because he provides too little effort compared to a rational player. This, in turn, discourages the other’s effort. Consequently, both effects lower the player’s payoff. In contrast, if the player is overconfident, the strategic effect is positive because the player provides too much effort, which encourages the other’s effort. In this case, whether the direct or the strategic effect dominates, depends on the extent of the player’s self-confidence bias. If his bias is small, the strategic effect dominates, and he is better off; while if his bias is large, the direct effect dominates, and he is worse off.

The final hypothesis concerns the effect of self-confidence biases on team payoff, i.e., team revenue minus the sum of individual costs of effort.

#### Hypothesis 6

Team payoff increases in self-confidence bias.

If an overconfident player exerts more effort, this extra effort directly reduces free-riding by this player and raises team payoffs. In addition, due to the complementarity between the players’ efforts, it also increases the other’s marginal productivity and, thus, the other’s effort. As a result, team payoffs raise even further. All six hypotheses are in line with the original model of Gervais and Goldstein (2007).

## Experiment

In this section, we present the experiment. We first describe its general structure and, subsequently, explain each of its four main blocks in detail.

### General structure

The experiment took place at the laboratory of the University of Lausanne (LABEX) in November 2019. It involved mostly students of various academic fields from the University of Lausanne and the École Polytechnique Fédérale de Lausanne (EPFL), whom we recruited from the subject pool of the LABEX via ORSEE (Greiner, 2015). Our two main treatments involved 240 subjects, 10 sessions, each comprising 24 subjects.^9^ In every session, we randomly assigned subjects into two *groups* of 12. Within each group, we randomly matched subjects into 6 *teams* of two players.

To test our hypotheses, the experiment features a between-subjects design with two main treatments. In these treatments, we exogenously manipulate the subjects’ belief regarding their ability multiplier by employing either an easy or a hard general knowledge quiz. Each team of two engages in an effort task. Half of the sessions expose subjects to the EASY treatment, while the other half exposes them to the HARD treatment.^10^

The experiment comprises four main blocks as shown in Table 1. We now describe the different blocks in more detail. Instructions for the experiment, including control questions, can be found in Appendix L.

**Table 1:** Main blocks of the experiment

Block 1	Belief manipulation & elicitation of prior beliefs
Block 2	Team effort task
Block 3	Elicitation of posterior beliefs, social & risk preferences, and demographics
Block 4	Payment

### Belief manipulation & elicitation of prior beliefs

In Block 1, subjects are first randomly assigned to the two treatments, EASY and HARD, which exogenously manipulate self-confidence using a general knowledge quiz. The quiz is based on Moore and Healy (2008) and comprises 46 questions which are divided into six different general knowledge topics: Science, Geography, Movies, Music, History, and Switzerland. Depending on the treatment, the questions are either easy or hard. Easy questions induce overconfidence and hard ones induce underconfidence due to the “hard-easy” effect in relative placement (Kruger, 1999; Moore & Kim, 2003; Moore & Cain, 2007; Moore & Healy, 2008; Dargnies et al., 2019). Subjects have 20 min to complete the quiz.

The number of correct answers determines a subject’s rank, $r_i$, within his group of twelve. The best performer gets a rank of $r_i=1$, while the worst performer gets a rank of $r_i=12$. Ties are broken randomly. A subject’s rank directly maps into his ability multiplier measured in tokens,

$$\begin{aligned} a_i = (13-r_i) \times 20 , \end{aligned}$$

which remains constant throughout the team effort task.^11^ For instance, the second best performer with rank $r_i=2$ gets an ability multiplier of $a_i=220$. Throughout the experiment, subjects are never told about their rank or ability multiplier.

Next, we elicit the subjects’ prior belief about their rank $\tilde{r}_i$. Subjects replied to the following request: “We wish you to provide us with your estimate of your rank as an integer between 1 and 12.” As subjects may report biased and inaccurate beliefs (Grether, 1992), we elicit beliefs in an incentive-compatible way using the binarized scoring rule by Hossain and Okui (2013). Under this scoring rule, subjects have an incentive to disclose their true belief, irrespective of their risk preferences, even if they are non-expected utility maximizers. The prior belief maps into the perceived ability multiplier, $\tilde{a}_i = (13-\tilde{r}_i) \times 20$, determining the self-confidence bias $b_i = \tilde{a}_i - a_i$. Notice that to mitigate strategic incentives to misreport beliefs, we elicited beliefs before the team effort task.^12^

### Team effort task

In Block 2, subjects were exposed to a team version of the ball catching task by Gächter et al. (2016). This effort task offers two main advantages. First, it ensures that all subjects face the same cost of effort, which can be defined by the experimenter. Second, effort provision has proven to be more sensitive to changes in incentives than in other common effort tasks (Gächter et al., 2016; Araujo et al., 2016).

**Fig. 1:**
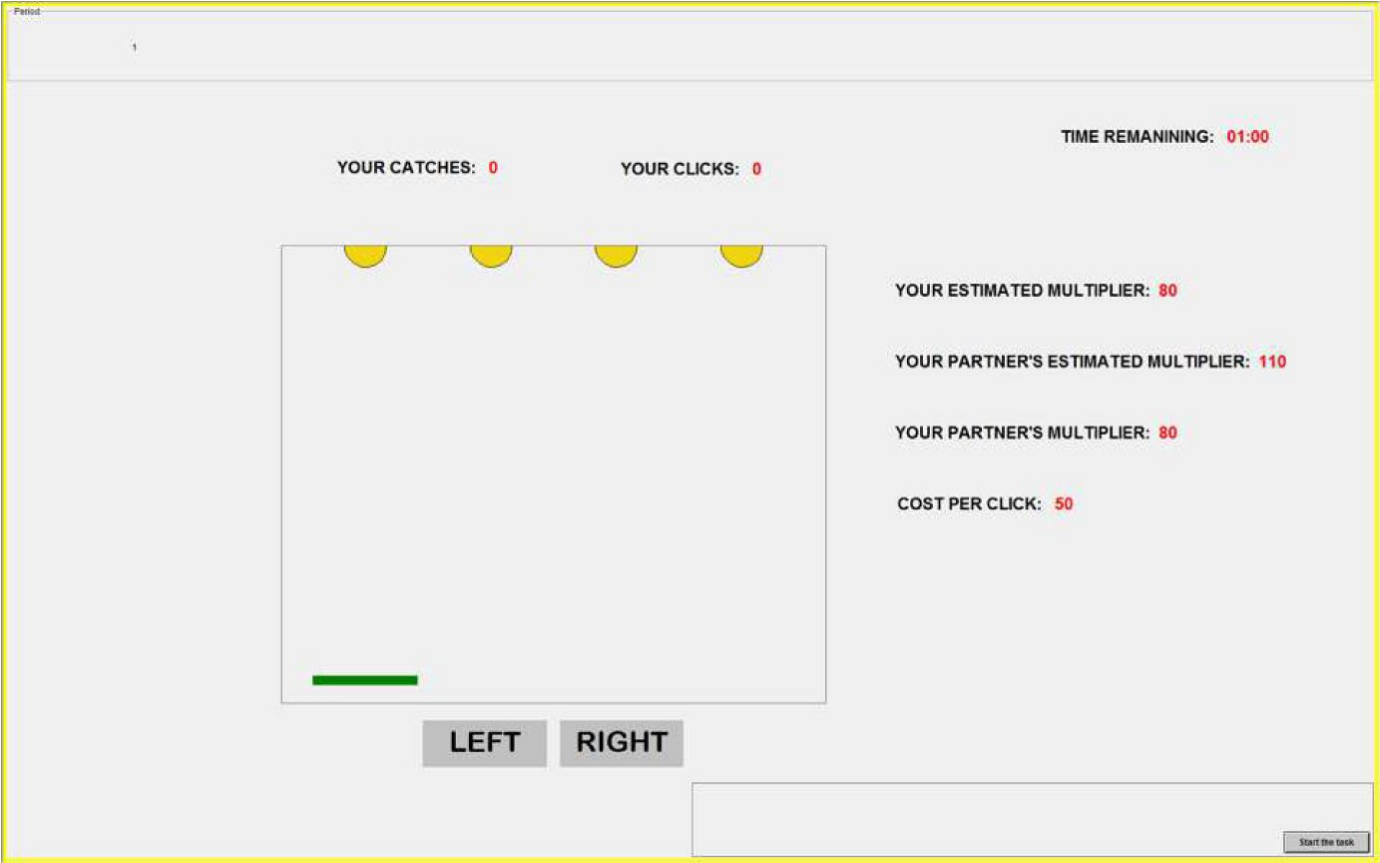
Interface of the Ball Catching Task

The interface of our version of the ball catching task is shown in Fig. 1. Balls randomly fall from one of the four positions at the top of the screen. Each subject has to catch them by moving the green tray at the bottom of the screen in order to earn tokens. A click on the buttons “LEFT” and “RIGHT” moves the tray by one position in the corresponding direction at a cost of *c* tokens per click. In addition, the screen provides the subject with the following information (in clockwise order): i) his number of catches and clicks; ii) the remaining time to complete the task; iii) his own perceived ability multiplier, $\tilde{a}_i$; iv) the other subject *j*’s perceived and true ability multipliers, $\tilde{a}_j$ and $a_j$, respectively; and v) the cost per click.

The subject’s ability multiplier and catches, $a_i$ and $q_i$, as well as the other’s ability multiplier and catches, $a_j$ and $q_j$, determine the team revenue in tokens as in Eq. (1). We set the team revenue parameter $w=1$, the effort complementarity parameter $s=5$, and the unit cost of clicks $c=50$ tokens. This parametrization, together with the range of the ability multipliers, creates a non-trivial trade-off between benefits and costs of effort where subjects with high ability multipliers have an incentive to click more to catch balls further away from the tray. The subject knows that his individual payoff and the other’s payoff will correspond to half of the team revenue minus the individual costs of effort.

Each team performs the ball catching task over eight periods. At the end of every period, each subject is informed about his number of catches, clicks, and individual payoff. To familiarize subjects with the task, there were also trial periods at the beginning, where each subject participated in the individual version of ball catching task of Gächter et al. (2016) with varying ability multipliers. We confirm that effort provision is sensitive to changes in incentives, i.e., changes in ability multipliers (see Appendix E).

Note that the absence of a relation between the general knowledge quiz and the ball-catching task enables us to cleanly identify the causal effect of self-confidence bias on teamwork. This mitigates potential confounding factors associated with using the same task to gauge both self-confidence and effort provision.

### Elicitation of posterior beliefs, social & risk preferences, and demographics

In Block 3, we elicit subjects’ posterior beliefs about their ranks, their social and risk preferences as well as some of their demographic characteristics.

Subjects can update their beliefs about their rank and state a posterior belief. We give them this option as the information about individual clicks, catches, and payoffs at the end of each period could lead to learning. This information represents a series of eight signals about a subject’s ability multiplier and, thus, her rank. However, the signals are noisy as the subject’s payoff in a given period also depends on the teammate’s catches. In case a subject updates her belief, there is a fifty-fifty chance that either the prior or the posterior belief counts for payment. In case the subject does not update her belief, the prior belief counts for payment.

For eliciting social preferences, we use the task by Balafoutas et al. (2012), which allows us to classify each subject either as efficiency-loving, inequality-averse, inequality-loving, or spiteful. For eliciting risk preferences, we apply the Bomb Risk Elicitation Task (BRET) by Crosetto and Filippin (2013), which is easy to understand and provides an individual index of risk aversion. We also asked subjects to provide the following demographics: age, gender, nationality, whether only child (0/1), parents’ educational attainment, number of acquaintances, whether living in a big town (0/1), being enrolled at the University of Lausanne (0/1), study program (Bachelor/Master), and GPA.

### Payment

In Block 4, each subject receives his payment. All payments occur in this final block to avoid any income effects. The total payment comprises the following five elements: (i) a show-up fee of CHF 5.00; (ii) a payment for relative performance in the general knowledge quiz in the group, ranging from CHF 0.20 to 2.40; (iii) a payment for the accuracy of the belief about the rank in the group, ranging from CHF 0.00 to 2.00; iv) a payment for a randomly selected period of the ball catching task, amounting to CHF 16.67, on average; v) a payment for the elicitation of the distributional and risk preferences, ranging from CHF 0.62 to 1.05 and from CHF 0 to 2.48, respectively. The average total payment is CHF 30.60 for a duration of approximately 90 min. The conversion rate is CHF 1.00 per 300 tokens. At the time of the experiment, CHF 1.00 was worth roughly USD 1.01.

## Results

In this section, we present the results. We first discuss the descriptive results of the belief manipulation. Subsequently, we show how the treatment affects effort via a shift in beliefs, and we turn to the regressions that test the hypotheses derived from the theory. Finally, we look into potential learning effects and delayed reactions to the teammate’s ability and self-confidence bias.

### Belief manipulation

**Fig. 2:**
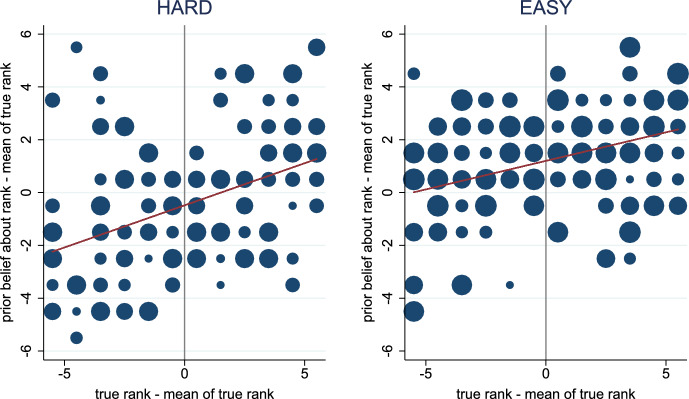
Relationship between Prior Beliefs and True Ranks. The horizontal axis displays the demeaned version of the subjects’ true ranks, $r_i-{\bar{r}}$, where ${\bar{r}}=6.5$. The vertical axis shows the difference between the subjects’ prior beliefs about their rank and the mean of the *true* ranks, $\tilde{r}_i - {\bar{r}}$. Regression lines are obtained from regressions based on the specification in Eq. (3). The size of the circles is proportional to the number of subjects represented by them

Figure  2 confirms that the belief manipulation succeeded. It exhibits the relationship between subjects’ true ranks and prior beliefs about their rank across the two treatments. The left panel shows this relationship for the HARD treatment while the right panel shows it for the EASY treatment.

The scaling of the axes ensures that the intercepts of the depicted regression lines reflect the subjects’ average level of overconfidence. That is, the horizontal axis displays the demeaned version of the subjects’ true ranks, $r_i-{\bar{r}}$, where ${\bar{r}}=6.5$. The vertical axis shows the difference between the subjects’ prior beliefs about their rank and the mean of the *true* ranks, $\tilde{r}_i - {\bar{r}}$. Hence, the regressions underlying the two depicted lines have the following specification:

3 $$\begin{aligned} \tilde{r}_i - {\bar{r}} = \alpha _0 + \alpha _1 \, (r_i-{\bar{r}}) + u_i \,. \end{aligned}$$

In this specification, the intercept indicates whether subjects are, on average, overconfident ($\alpha _0>0$) or underconfident ($\alpha _0<0$). The slope reflects whether their beliefs are precise ($\alpha _1$ close to 1) or noisy ($\alpha _1$ close to zero).

The first finding confirms that the belief manipulation is effective. In the HARD treatment, subjects underestimate their ranks (${\hat{\alpha }}_0=-0.483$; two-sided *t*-test: *p*-value=0.022); while in the EASY treatment, they overestimate their ranks (${\hat{\alpha }}_0=1.200$; two-sided *t*-test: *p*-value<0.001).

The second finding reveals that prior beliefs react to true ranks and that there is substantial noise in prior beliefs. In the HARD treatment ${\hat{\alpha }}_1 = 0.319$ (two-sided *t*-test: *p*-value<0.001), while in the EASY treatment ${\hat{\alpha }}_1 = 0.216$ (two-sided *t*-test: *p*-value < 0.001).^13^ Prior beliefs predict R^2^=19.0% of the variance in true ranks in the HARD treatment and R^2^=14.3% in the EASY treatment.

Although the mechanism through which our treatment, EASY vs. HARD quiz, changes subjects’ beliefs about their ability is not the main scope of the paper, there is an interesting similarity to the work by Butler (2016). In an experiment that randomly assigns inequality in payments, he shows that such inequality causes the advantaged to display higher beliefs about their relative ability than the disadvantaged. In contrast, in our experiment, inequality in payments is not salient as we do not directly manipulate earnings. Yet, subjects exposed to an EASY quiz may still perceive themselves to be in an advantageous position relative to others, which could be the reason behind their higher self-confidence. Hence, an avenue for future research is to investigate whether inequality in opportunities influences self-confidence even in settings, such as ours, where it is not salient.^14^

### Treatment effects through shifts in beliefs

**Fig. 3:**
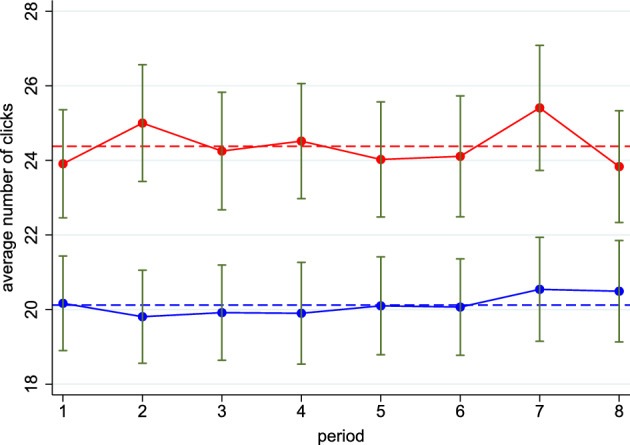
Average Efforts across Periods and Treatments. The treatment HARD is depicted in blue, and the treatment EASY in red. Solid lines show the average effort per period, while dashed lines show the average effort over all periods. Standard errors (in green) are clustered at the team level

Figure 3 reveals that, across all eight periods, the average effort per period in the HARD treatment falls consistently short of the one in the EASY treatment. Subjects provide an average effort per period of 20.12 clicks in the HARD treatment and 24.38 clicks in the EASY treatment (dashed lines), corresponding to a difference of 21.1% (two-sided z-test from a Generalized Least Squares (GLS) regression: *p*-value = 0.020).

Next, we analyze whether the observed difference in effort across treatments is due to differences in beliefs about ability multipliers. Table 2 exhibits the results of three Generalized Least Squares (GLS) regressions, where the dependent variable is always effort in clicks. The regressions feature random effects to account for the ball-catching task’s stochastic production function, where the marginal revenue of a click depends on the random order of the falling balls. Standard errors are clustered at the team level. The specification in Column (1) confirms the average treatment difference in effort across all periods discussed above. The specification in Column (2) controls for the subjects’ true ability multipliers as well as gender. It shows that men provide, on average, 9.85 clicks more than women and that the treatment difference remains significant. The specification in Column (3) adds the subjects’ perceived ability multipliers. It reveals that, once we control for the focal subject *i*’s perceived ability multiplier the treatment dummy gets much smaller in size and becomes insignificant. Thus, the treatment indeed affects beliefs: subjects exert more effort in the EASY treatment because they are more confident regarding their ability multiplier.

**Fig. 4:**
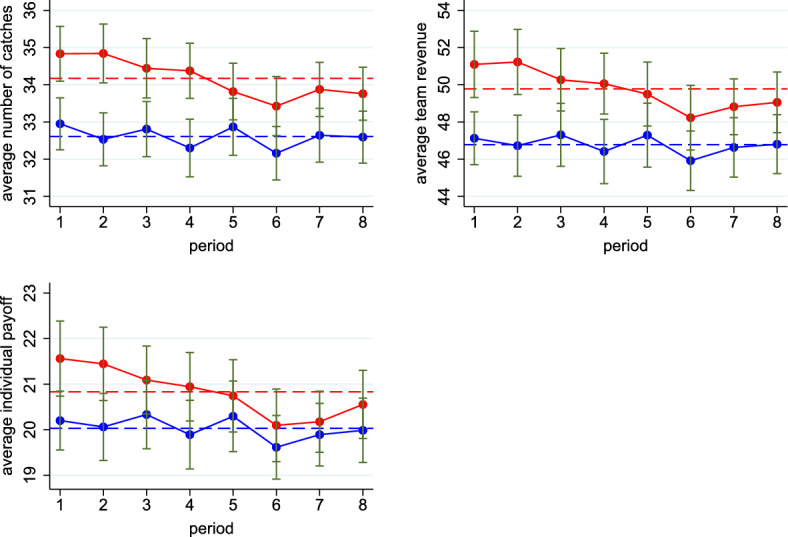
Catches, Team Revenue, and Payoffs across Periods and Treatments. The treatment HARD is depicted in blue, and the treatment EASY in red. Solid lines show the average catches, team revenue, and individual payoff per period. Dashed lines display the average catches, team revenue, and individual payoff over all eight periods. Standard errors (in green) are clustered at the team level

Moreover, Figure 4 shows additional differences between treatments. In particular, it reveals that moving from the HARD to the EASY treatment raises the number of catches by 4.8%, team revenue by 6.4%, and average individual payoffs by 4.0%. Although these differences are not statistically significant, they go in the expected direction and their sizes are large. Note that over the eight periods, there is a decline in catches, team revenue, and individual payoffs in the EASY treatment. This decline cannot be due to learning about own ability since that would imply a change in the average number of clicks (which, as we saw in Fig. 3, remains constant). Hence, this decline is most likely due to subjects’ complacency or fatigue in the EASY treatment.

Since we test the significance of differences in outcomes across four dimensions in the same sample, we adjust for multiple hypotheses testing (List et al., 2019). Appendix F shows the corresponding adjusted *p*-values and reveals that the main result regarding effort, measured as the number of clicks, remains significant. Hence, the treatments exogenously shift subjects’ beliefs about their ability multipliers and result in a significantly different effort provision.^15^

**Table 2:** Treatment, beliefs, and effort provision

Effort in clicks	(1)	(2)	(3)
Constant	20.1240^***^	9.3452^***^	2.1390^***^
	(1.2019)^***^	(2.5603)^***^	(3.4583)^***^
EASY	4.2573^***^	2.8621^***^	0.0531^***^
	(1.8316)^***^	(1.6765)^***^	(1.8404)^***^
*i*’s ability multiplier ($a_{i}$)		0.0153^***^	0.0063^***^
		(0.0133)^***^	(0.0141)^***^
*j*’s ability multiplier ($a_{j}$)		0.0323^***^	0.0317^***^
		(0.0130)^***^	(0.0135)^***^
Male		9.8481^***^	8.7378^***^
		(1.6631)^***^	(1.6852)^***^
*i*’s perceived ability multiplier ($\tilde{a}_{i}$)			0.0871^***^
			(0.0230)^***^
*j*’s perceived ability multiplier ($\tilde{a}_{j}$)			0.0011^***^
			(0.0188)^***^
No. of observations	1,920^***^	1,920^***^	1,920^***^
R^2^	0.0179^***^	0.1366^***^	0.1867^***^

The table reports the results of random-effects GLS regression with the number of clicks as dependent variable and a dummy for the EASY treatment as the main regressor. Standard errors are clustered at the team level. Significantly different from zero at 1% (^***^), 5% (^**^), 10% (^*^)

### Test of model hypotheses

We now test Hypotheses 1 and 2 using a random-effects GLS regression on effort provision with the following specification:

4 $$\begin{aligned} e_{it} = \beta _0 + \beta _1 a_i + \beta _2 b_i + \beta _3 a_j + \beta _4 b_j + \beta '_5 X_i + \beta '_6 P_t + u_{it} . \end{aligned}$$

The dependent variable is the effort of subject *i* in period *t*, $e_{it}$, measured in clicks. $X_i$ is a vector comprising *i*’s social and risk preferences as well as demographic characteristics, $P_t$ is a vector of period dummies, and $u_{it}$ is the stochastic error term. In this specification, each subject in a team appears twice, once as the focal individual *i* and once as the teammate *j*. To take this into account, standard errors are clustered at the team level.^16^

To test Hypothesis 3, we use a random-effects GLS regression on team revenue with the following specification:

5 $$\begin{aligned} R_{ijt} = \gamma _0 + \gamma _1 a_i + \gamma _2 b_i + \gamma _3 a_j + \gamma _4 b_j + \gamma '_5 X_{ij} + \gamma _6' P_t + u_{ijt} . \end{aligned}$$

The dependent variable is the team revenue of subjects *i* and *j* in period *t*, $R_{ijt}$, measured in CHF. In this specification, *i* always represents the subject with the higher ability multiplier relative to the teammate *j*, i.e., $a_i > a_j$. $X_{ij}$ contains team-specific controls, while $P_t$ is the vector of period dummies as before. Notice that by including ability multipliers as well as self-confidence biases, any correlation between these variables is taken into account.

**Table 3:** Effort and team revenue regressions

	Effort in Clicks ($e_{it}$)			Team Revenue in CHF ($R_{ijt}$)		
constant	${\hat{\beta }}_0$	3.0259^***^	1.1244^***^	${\hat{\gamma }}_0$	-1.7254^***^	5.8524^***^
		(3.5799)^***^	(8.6476)^***^		(4.3806)^***^	(15.0389)^***^
*i*’s ability	${\hat{\beta }}_1$	0.1065^***^	0.0718^***^	${\hat{\gamma }}_1$	0.2058^***^	0.1722^***^
multiplier ($a_{i}$)		(0.0199)^***^	(0.0213)^***^		(0.0256)^***^	(0.0266)^***^
*i*’s self-confidence	${\hat{\beta }}_2$	0.1042^***^	0.0871^***^	${\hat{\gamma }}_2$	0.0348^***^	0.0300^***^
bias ($b_i$)		(0.0230)^***^	(0.0213)^***^		(0.0214)^***^	(0.0209)^***^
*j*’s ability	${\hat{\beta }}_3$	0.0351^***^	0.0396^***^	${\hat{\gamma }}_3$	0.1729^***^	0.1772^***^
multiplier ($a_j$)		(0.0186)^***^	(0.0176)^***^		(0.0206)^***^	(0.0210)^***^
*j*’s self-confidence	${\hat{\beta }}_4$	0.0089^***^	0.0028^***^	${\hat{\gamma }}_4$	0.0718^***^	0.0521^***^
bias: ($b_j$)		(0.0186)^***^	(0.0181)^***^		(0.0252)^***^	(0.0236)^***^
Controls (*X* and *P*)		no^***^	yes^***^		no^***^	yes^***^
No. of observations		1,920^***^	1,920^***^		960^***^	960^***^
R^2^		0.1182^***^	0.2497^***^		0.6456^***^	0.7139^***^

The table shows the results of Regressions (4) and (5) with and without controls. These are GLS regressions with random effects and standard errors clustered at the team level. A version of the table showing all estimates, including the ones for the controls, is in Appendix I. Significantly different from zero at 1% (^***^), 5% (^**^), 10% (^*^)

Table 3 exhibits the results of the two regressions. We start with the first regression on effort provision. The estimate ${\hat{\beta}}_2$ indicates that subject *i*’s own self-confidence bias has a highly significant and positive effect on his effort. That is, a unit increase in *i*’s self-confidence bias increases his effort by 0.10 clicks on average. This first result confirms Hypothesis 1. To check the robustness of Result 1, we analyzed subjects’ behavior separately in period 1 and all following periods. Details can be found in Appendix K. Overall, our results remain robust. Moreover, the analysis of period 1 behavior suggests that the information disclosed during the first period reinforced subjects’ understanding that they are in a team setting and that, on average, teammates with higher ability multipliers are more productive.

#### Result 1

Subject *i*’s self-confidence bias has a positive effect on his effort.

The estimate ${\hat{\beta }}_4$ reveals that the other subject *j*’s self-confidence bias has no significant effect on *i*’s effort. This second result does not support Hypothesis 2.

#### Result 2

The other subject *j*’s self-confidence bias has no significant effect on the focal subject *i*’s effort.

However, even though subjects do not react to the other’s self-confidence bias, they do react to the other’s ability multiplier as shown by the positive and significant estimate ${\hat{\beta }}_3$. Thus, subjects understand that they are in a team where, in equilibrium, the marginal returns to effort increase in the other’s ability multiplier.

Overall, Results 1 and 2 together imply that the first channel –relying on effort and ability being complements– accounts for most of the positive effect of overconfidence on increasing effort and reducing free-riding. At the same time, there is no evidence for the second channel, relying on a strategic reaction to the perceived overconfidence of the teammate.

We now turn to the second regression on team revenue to test Hypothesis 3. The estimates ${\hat{\gamma }}_3$ and ${\hat{\gamma }}_4$ show that team revenue significantly increases in *j*’s ability mutliplier and self-confidence biases. A unit increase in the low-ability subject *j*’s ability multiplier raises the average team revenue by CHF 0.17, while a unit increase in the low-ability subject *j*’s self-confidence bias raises the average team revenue by CHF 0.07. This third result confirms Hypothesis 3.

#### Result 3

Team revenue increases in self-confidence bias.

To test Hypotheses 4 and 5, we estimate the following random-effects GLS regression on individual payoffs:

6 $$\begin{aligned} U_{it} = \delta _0 + \delta _1 a_i + \delta _2 b_i + \delta _3 b_i^2 + \delta _4 a_j + \delta _5 b_j + \delta '_6 X_i + \delta '_7 P_t + u_{it} . \end{aligned}$$

The dependent variable is the payoff of subject *i* in period *t*, $U_{it}$, measured in CHF. Apart from the quadratic form of the self-confidence bias to test Hypothesis 5 – i.e., that a player’s payoff is inversely u-shaped in his self-confidence bias – the specification is analogous to the one on effort.

To test Hypothesis 6, we estimate the following random-effects GLS regression on team payoff:

7 $$\begin{aligned} \pi _{ijt} = \eta _0 + \eta _1 a_i + \eta _2 b_i + \eta _3 a_j + \eta _4 b_j + \eta '_5 X_{ijt} + \eta _6' P_t + u_{ijt} , \end{aligned}$$

where the dependent variable $\pi _{ijt}=R_{ijt}-ce_{it}-ce_{jt}$ is team payoff, measured in CHF. As in Regression (5), *i* is the subject with the relatively higher ability multiplier in the team, $X_{ijt}$ denotes team-specific controls, and $P_t$ represents period dummies.

**Table 4:** Payoff regressions

	Individual payoff			Team payoff		
	in CHF ($U_{it}$)			in CHF ($\pi _{ijt}$)		
Constant	${\hat{\delta }}_0$	−0.6219^***^	3.3487^***^	${\hat{\eta }}_0$	−2.0243^***^	2.3941^***^
		(1.3575)^***^	(3.3344)^***^		(3.3936)^***^	(11.2861)^***^
*i*’s ability	${\hat{\delta }}_1$	0.0770^***^	0.0747^***^	${\hat{\eta }}_1$	0.1747^***^	0.1497^***^
Multiplier ($a_i$)		(0.0055)^***^	(0.0061)^***^		(0.0195)^***^	(0.0199)^***^
*i*’s self-confidence	${\hat{\delta }}_2$	0.0110^***^	0.0089^***^	${\hat{\eta }}_2$	0.0161^***^	0.0143^***^
bias ($b_i$)		(0.0055)^***^	(0.0061)^***^		(0.0172)^***^	(0.0164)^***^
*i*’s self-confidence	${\hat{\delta }}_3$	−0.0001^***^	−0.0001^***^			
Bias squared ($b_i^2$)		(0.0001)^***^	(0.0001)^***^			
*j*’s ability	${\hat{\delta }}_4$	0.0862^***^	0.0870^***^	${\hat{\eta }}_3$	0.1550^***^	0.1587^***^
Multiplier ($a_j$)		(0.0059)^***^	(0.0061)^***^		(0.0145)^***^	(0.0143)^***^
*j*’s self-confidence	${\hat{\delta }}_5$	0.0222^***^	0.0225^***^	${\hat{\eta }}_4$	0.0501^***^	0.0346^***^
Bias: ($b_j$)		(0.0058)^***^	(0.0060)^***^		(0.0183)^***^	(0.0170)^***^
*p*-value of Wald test for						
joint significance of $b_i$ and $b_i^2$		0.0497^***^	0.2270^***^			
Controls (*X* and *P*)		no^***^	yes^***^		no^***^	yes^***^
No. of observations		1,920^***^	1,920^***^		960^***^	960^***^
R^2^		0.6823^***^	0.7006^***^		0.7155^***^	0.7642^***^

The table shows the results of Regressions (6) and (7) with and without controls. These are GLS regressions with random effects and standard errors clustered at the team level. A version of the table showing all estimates, including the ones for the controls, is in Appendix I. Significantly different from zero at 1% (^***^), 5% (^**^), 10% (^*^)

Table 4 exhibits the results of Regressions (6) and (7). The estimate ${\hat{\delta }}_4$ confirms that the other subject *j*’s self-confidence bias has a positive and significant effect on subject *i*’s payoff. A unit increase in *j*’s self-confidence bias raises *i*’s payoff, on average, by CHF 0.09. This result confirms Hypothesis 4.

#### Result 4

The other subject *j*’s self-confidence bias raises the focal subject *i*’s payoff.

**Fig. 5:**
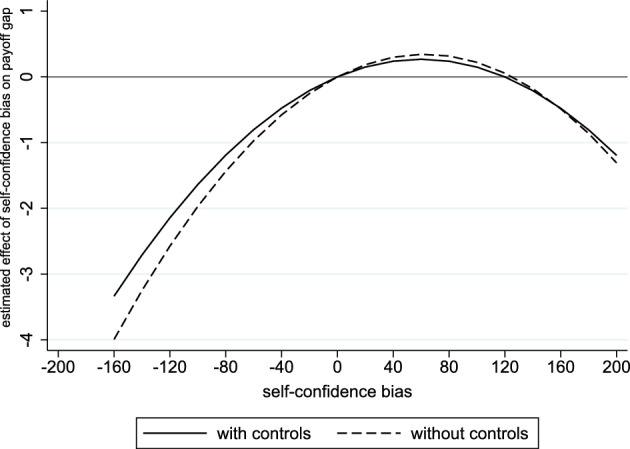
Self-Confidence Bias and Payoff. The figure displays the predicted payoff gap in CHF relative to an unbiased subject with the same ability, teammate, and characteristics. The estimated shape is based on Regression (6)

The estimates ${\hat{\delta }}_2$ and ${\hat{\delta }}_3$ confirm that a subject’s self-confidence bias has an inverse u-shaped effect on his payoff. The estimates are significant in the version without controls (Wald test for joint significance: *p*-value = 0.050) but, due to the larger standard errors, become insignificant once we add controls (Wald test for joint significance: *p*-value = 0.227). Figure 5 illustrates the result by showing how the self-confidence bias affects the payoff gap relative to an unbiased subject with the same ability, teammate, and characteristics. This result supports Hypothesis 5.

#### Result 5

A subject’s self-confidence bias has an inversely u-shaped effect on his payoff.

The estimates ${\hat{\eta }}_2$ and ${\hat{\eta }}_4$ reveal that a subject’s self-confidence bias has a positive effect on team payoff, although only the coefficient for the high-ability subject *i* is statistically significant. This result confirms the final Hypothesis 6.

#### Result 6

Team payoff increases in teammates’ self-confidence biases.

One potential concern is that our results may be primarily driven by mood effects. If the EASY quiz induced a positive mood and the HARD quiz a negative one, and a better mood led subjects to exert more effort, then the difference in moods could be responsible for the higher effort in the EASY treatment. To rule out this alternative explanation we conducted two additional treatments. These treatments replicate the original ones, including belief elicitation about quiz ranking, but we let subjects know their true ability multipliers when they perform the team effort task. This allows us to test whether mood effects play a role as we rule out self-confidence effects. The results reveal that mood effects only play a minor role. The average effort in the EASY treatment is only 8.5% higher than in the HARD treatment, but this difference is not statistically significant.^17^

### Potential learning and delayed reaction to the teammate’s ability and self-confidence bias

We now look into potential learning effects with regard to subjects’ own ability and delayed reaction to the teammate’s ability and self-confidence bias.

#### Learning with Regard to Own Ability

As discussed in Sect. 3.4, after performing the team effort task in Block 2, we gave subjects the option to update their belief about their rank. The aim was to check whether there was any systematic decline in self-confidence biases over the eight periods due to learning.

Overall, 113 out of the 240 subjects updated their beliefs. In the HARD treatment, 62 subjects made an update, while in the EASY treatment, just 51 updated their beliefs. This difference is not statistically significant (two-sided *t*-test: *p*-value = 0.156).

**Fig. 6:**
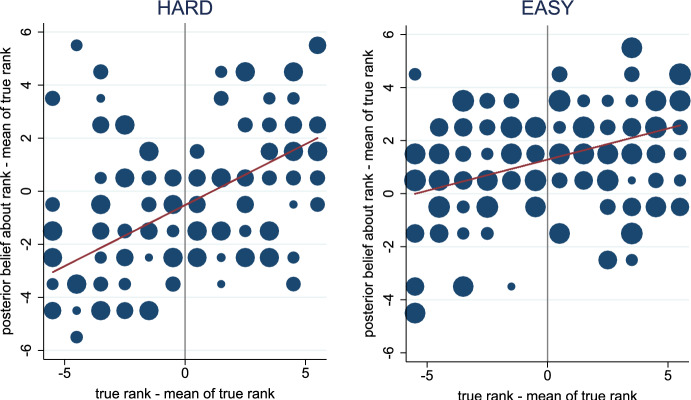
Relationship between Posterior Beliefs and True Ranks. The horizontal axis displays the demeaned version of the subjects’ true ranks, $r_i-{\bar{r}}$, where ${\bar{r}}=6.5$. The vertical axis shows the difference between the subjects’ posterior beliefs about their rank and the mean of the *true* ranks, $\tilde{r}_i - {\bar{r}}$. Notice that for subjects who did not update their belief, the posterior equals the prior belief. Regression lines are obtained from regressions based on the specification in Eq. (3), where we replace prior with posterior beliefs. The size of the circles is proportional to the number of subjects represented by them

Figure 6 shows how posterior beliefs relate to ranks. It is analogous to Fig. 2 but uses posterior instead of prior beliefs. The belief manipulation is still effective as, on average, subjects underestimate their rank in the HARD treatment (${\hat{\alpha }}_0=-0.525$; two-sided *t*-test: *p*-value = 0.008) and overestimate it in the EASY treatment (${\hat{\alpha }}_0=1.283$; two-sided *t*-test: *p*-value < 0.001). Moreover, posterior beliefs react to true ranks but there is substantial noise. In the HARD treatment ${\hat{\alpha }}_1 = 0.460$ (two-sided *t*-test: *p*-value < 0.001), while in the EASY treatment ${\hat{\alpha }}_1 = 0.234$ (two-sided *t*-test: *p*-value < 0.001).

**Table 5:** Potential delay in effort

	Effort in Clicks ($e_{it}$)	
Constant	3.8004^***^	2.1140^***^
	(3.5972)^***^	(8.6436)^***^
*i*’s ability	0.1065^***^	0.0718^***^
Multiplier ($a_i$)	(0.0199)^***^	(0.0212)^***^
*i*’s self-confidence	0.1042^***^	0.0871^***^
Bias ($b_i$)	(0.0230)^***^	(0.0213)^***^
Period counter ($p_t$)	0.1721^***^	0.1721^***^
	(0.2888)^***^	(0.2900)^***^
*j*’s ability	0.0284^***^	0.0329^***^
Multiplier ($a_j$)	(0.0191)^***^	(0.0182)^***^
$a_j \times p_t$	0.0015^***^	0.0015^***^
	(0.0019)^***^	(0.0019)^***^
*j*’s self-confidence	0.0045^***^	0.0106^***^
Bias: ($b_j$)	(0.0207)^***^	(0.0197)^***^
$b_j \times p_t$	0.0030^***^	0.0030^***^
	(0.0022)^***^	(0.0022)^***^
*p*-value of Wald test for joint		
significance of $p_t$, $a_j \times p_t$ and $b_j \times p_t$	0.5270^***^	0.5305^***^
Controls (*X* and *P*)	no^***^	yes^***^
No. of observations	1920^***^	1920^***^
R^2^	0.1187^***^	0.2498^***^

The table shows the results of the random-effects GLS regression with and without controls. Standard errors are clustered at the team level. Significantly different from zero at 1% (^***^), 5% (^**^), 10% (^*^)

In sum, the average self-confidence bias (indicated by the intercept) remains virtually unchanged in both treatments. However, at the individual level, there is evidence for some learning in the HARD treatment but not in the EASY treatment. In the HARD treatment, posterior beliefs explain R = 36.6% of the variance in ranks, whereas prior beliefs only explain R^2^ = 19.0%. In the EASY treatment, the two percentages are nearly identical and amount to R^2^ = 15.6% and R^2^ = 14.3%, respectively.

#### Delayed Reaction to the Teammate’s Ability and Self-Confidence Bias

Next, we look into whether subjects’ effort reacts in a delayed manner to the information about the teammate’s ability and self-confidence bias. To do so, we re-estimate a version of the random-effects GLS Regression (4), which interacts with the teammate’s ability multiplier, $a_j$, and her self-confidence bias, $b_j$, each with a period counter, $p_t \in \{1,2,\ldots ,8 \}$. We replace the period dummies with the period counter to keep the model parsimonious and get a linear approximation of the potential delay in the subjects’ effort in clicks.

Table 5 exhibits the results. There is no evidence of a delayed reaction in subjects’ effort, neither with respect to the teammate’s ability nor with respect to her self-confidence bias. The corresponding coefficients are all insignificant, both individually and jointly (*p*-values of Wald tests for joint significance: *p* = 0.527 for the regression without controls, *p* = 0.531 for the regression with controls)

## Conclusion

Our findings have direct implications for setting up and managing teams. While worker overconfidence can have many negative consequences, we point out one way in which it can be beneficial. Our main finding, that overconfidence leads to more effort and less free riding, indicates that organizations could benefit from setting up overconfident teams and promoting overconfidence among their existing members. At the same time, the lack of evidence for the second channel, which relies on the perceived overconfidence of teammates, suggests that a strategy whereby workers signal own overconfidence to get their teammates to exert more effort is likely bound to fail. Similarly, a team leader making her subordinates aware of the prevailing overconfidence in the team may not result in higher effort.

In addition, the findings are economically significant. Subjects in the EASY treatment provide 21.1% more effort and catch 4.8% more balls than subjects in the HARD treatment. This increase in effort provision and productivity in the EASY treatment translates into a 6.4% increase in team revenue. Furthermore, our estimates indicate that, if a subject’s overconfidence increases by 3 ranks (i.e., roughly one standard deviation), his effort increases by 6.25 clicks, which corresponds to 28.1% of the average effort in our task. According to the estimates of team revenue, this increase in effort would result in an average increase in team revenue by 9.3%. Hence, setting up overconfident teams could lead to meaningful gains in team output.

There is also room for future research. The paper empirically tests the model of Gervais and Goldstein (2007), which rests on two main assumptions regarding teamwork. The first assumption is that players’ efforts are strategic complements. This first assumption finds empirical support in Friebel et al. (2017) who show in a field experiment in a large retail chain that complementarities in workers’ efforts are a feature of teamwork. The second assumption is that a player’s ability and effort are complements. For instance, in tasks where time (effort) and cognitive skills (ability) matter, a more able employee will produce higher output in the same time than a less able colleague (Sautmann, 2013). There is less empirical support for this assumption. Chen and Schilberg-Hörisch (2019) show in a laboratory experiment that ability and effort are complements, however the experiment is on individual and not on team effort. Ultimately, the validity of this assumption hinges on whether, in the trade-off between leisure and compensation, the substitution or the income effect dominates. Although extensively investigated in labor market literature with mixed results (for an overview, see Keane, 2011, 2022), to the best of our knowledge, this question has not yet been empirically examined within the context of teamwork.

Finally, potential detrimental effects of overconfidence, such as excessive risk-taking, intimidation of colleagues, and other negative effects on corporate culture, are beyond the scope of the paper. Future research could also investigate whether the findings are robust or whether they change if subjects know certain characteristics of their teammates, such as their gender or age.

## Supplementary Information

Below is the link to the electronic supplementary material.

Supplementary file 1 (pdf 851 KB)

## Footnotes

1 Charness et al. (2018) show that real effort tasks are better at measuring effort provision than hypothetical effort tasks when the potential source of variation is a psychological aspect, as in this paper.

2 Moore and Healy (2008) distinguish between three types of overconfidence: overestimation of one’s skill (absolute overconfidence), overplacement (relative overconfidence), and excessive precision in one’s beliefs (miscalibration or overprecision). Our study uses over- and underplacement in the general knowledge quiz to cause subjects to over- and underestimate their ability in the team effort task.

3 The pace at which the balls fall does not pose a challenge to subjects’ motor skills.

4 In a real world setting where teammates interact repeatedly, colleagues can observe signals of an individual’s confidence and true ability. For example, listening to others and openness to criticism are negatively correlated with overconfidence, while being persuasive and having high standards of individual and team performance are positively correlated with overconfidence (Kaplan et al., 2022) Furthermore, observing someone’s performance repeatedly reveals the person’s ability to the teammates. When someone’s confidence surpasses his performance systematically, the teammates will infer the person is overconfident. Our experimental design, by revealing the teammate’s ability and self-confidence bias in the first period, bypasses this process.

5 A robustness check, detailed in Appendix J.

6 Even though a subject’s effort does not react to the teammate’s self-confidence bias, it does react to the teammate’s ability. Thus, subjects understand that they are in a team where the marginal returns to equilibrium effort increase in the teammate’s ability.

7 Unpublished work by a partly overlapping set of co-authors looks into how perceptions of skill influence teamwork using a hypothetical effort task and without exogenously manipulating beliefs (Vialle et al., 2011).

8 In Appendix A, we show the analysis generalizes for a sufficiently concave $q(e_i)$.

9 The experiment involves two additional treatments to rule out mood effects which are described in Appendix J.

10 Descriptive statistics on the distribution of the social and risk preferences as well as the demographics are in Appendix D. They reveal that the randomization worked in most dimensions.

11 In the instructions, we present this mapping as a table containing all possible ranks and their corresponding ability multipliers.

12 To avoid any potential forms of deception, subjects were provided with the full set of instructions at the start of the experiment. This opens one potential way for strategic incentives to misreport beliefs to arise. Namely, if subjects realize that their teammate will later observe their true and perceived ability during the team effort task, subjects may overstate their perceived ability to make the teammate exert more effort. However, we believe this is highly unlikely for several reasons. First, the instructions are long (see Appendix L). Second, in the two additional treatments to detect potential mood effects, subjects have no incentive to strategically misreport their beliefs as the true ability multipliers of the two team members were disclosed to both before the start of the team effort task. Despite that, there is no significant difference in the levels of self-confidence biases in these additional treatments and the corresponding main treatments in the paper (see Appendix J for further details). Third, by overstating their belief, subjects would trade off a certain lower payment in the belief elicitation task for an uncertain higher payment in the team effort task. We find no evidence that risk-tolerant subjects overstate their beliefs more than risk-averse subjects (again, see Appendix J for further details). We thank an anonymous referee for pointing this out.

13 A joint regression with a treatment dummy on the intercept and slope parameters confirms that the two slopes are not significantly different (two-sided *t*-test: *p*-value = 0.189).

14 We thank an anonymous referee for highlighting this similarity.

15 Figures ?? and ?? in Appendix G depict the distributions of clicks and catches in each treatment. They reveal that the distributions of clicks and catches are more skewed to the left in the HARD than in the EASY treatment.

16 As a robustness check, and to be even closer to the theoretical equilibrium in Eq. (2), we also ran Regression (4) using the square root of effort as the dependent variable. Results remain unchanged as can be seen in Appendix H. However, in the main text we stick to the linear specification as the square root of individual effort may not exactly reflect the concavity of $q(e_i)$.

17 Appendix J describes the details of these additional treatments.
