# Supplementary material for: The role of self-confidence in teamwork: experimental evidence
Source: Exp Econ. 2024 May 18;27(3):687–712. doi: 10.1007/s10683-024-09829-x (PMC11637008; doi:10.1007/s10683-024-09829-x)
Supplement: Supplementary file 1 — (pdf 851 KB) [file 10683_2024_9829_MOESM1_ESM.pdf]

Appendix for Paper: “The Role of Self-Confidence in Teamwork: Experimental Evidence”

Adrian Bruhin Fidel Petros Luís Santos-Pinto*

May 1, 2024

*Authors affiliations: AB and LSP: University of Lausanne, Faculty of Business and Economics (HEC Lausanne), FP: WZB Berlin and TU Berlin; Corresponding author: Luís Santos-Pinto, Bâtiment Internef 535, University of Lausanne, CH-1015 Lausanne, Switzerland; luispedro.santospinto@unil.ch

A Generalized Model with a Concave $q(e)$ Function

In this appendix we show that the comparative static results of the theory model extend to a general concave $q(e)$ function:

$$q(e_i) = \alpha + \beta e_i^{\gamma},\ i = 1, 2, \tag{A.1}$$

where $\alpha, \beta > 0$ and $\gamma \in (0, 1/2]$. Note that the theory model in the paper assumes $\alpha = 0$, $\beta = 1$, and $\gamma = 1/2$. From (A.1) we have

$$q'(e_i) = \beta\gamma e_i^{\gamma-1},\ i = 1, 2, \tag{A.2}$$

and

$$q''(e_i) = -(1-\gamma)\beta\gamma e_i^{\gamma-2},\ i = 1, 2. \tag{A.3}$$

The perceived team revenue of player i is

$$\tilde{R}_i = 2w(\tilde{a}_1 q(e_1) + a_2 q(e_2) + sq(e_1)q(e_2)),\ i = 1, 2.$$

The perceived payoff of player i is

$$\tilde{U}_i = w(\tilde{a}_1 q(e_1) + a_2 q(e_2) + sq(e_1)q(e_2)) - ce_i,\ i = 1, 2.$$

The first-order conditions of players 1 and 2 are

$$\begin{aligned} wq'(e_1)\left[\tilde{a}_1 + sq(e_2)\right] - c &= 0 \\ wq'(e_2)\left[\tilde{a}_2 + sq(e_1)\right] - c &= 0 \end{aligned}$$

or

$$\begin{aligned} wq'(e_1)\left[(a_1 + b_1) + sq(e_2)\right] - c &= 0 \\ wq'(e_2)\left[(a_2 + b_2) + sq(e_1)\right] - c &= 0 \end{aligned}$$

Differentiation of the first-order conditions with respect to e_1, e_2, and b_1 gives us:

$$\begin{aligned} wq''(e_1)\frac{\partial e_1}{\partial b_1}\left[(a_1 + b_1) + sq(e_2)\right] + wq'(e_1)\left[1 + sq'(e_2)\frac{\partial e_2}{\partial b_1}\right] &= 0 \\ wq''(e_2)\frac{\partial e_2}{\partial b_1}\left[(a_2 + b_2) + sq(e_1)\right] + swq'(e_2)q'(e_1)\frac{\partial e_1}{\partial b_1} &= 0 \end{aligned}$$

Solving the first-order condition of player 2 with respect to $\partial e_2/\partial b_1$ we obtain

$$\frac{\partial e_2}{\partial b_1} = -\frac{sq'(e_2)q'(e_1)}{q''(e_2)\left[(a_2 + b_2) + sq(e_1)\right]}\frac{\partial e_1}{\partial b_1}. \tag{A.4}$$

Substituting this equation into the first-order condition of player 1 we obtain

$$q''(e_1)\frac{\partial e_1}{\partial b_1}\left[(a_1+b_1)+sq(e_2)\right]+q'(e_1)\left[1-\frac{s^2[q'(e_2)]^2q'(e_1)}{q''(e_2)\left[(a_2+b_2)+sq(e_1)\right]}\frac{\partial e_1}{\partial b_1}\right]=0,$$

or

$$q''(e_1)\frac{\partial e_1}{\partial b_1}\left[(a_1+b_1)+sq(e_2)\right]+q'(e_1)-\frac{s^2[q'(e_2)]^2[q'(e_1)]^2}{q''(e_2)\left[(a_2+b_2)+sq(e_1)\right]}\frac{\partial e_1}{\partial b_1}=0,$$

or

$$\frac{\partial e_1}{\partial b_1}\left[q''(e_1)\left[(a_1+b_1)+sq(e_2)\right]-\frac{s^2[q'(e_2)]^2[q'(e_1)]^2}{q''(e_2)\left[(a_2+b_2)+sq(e_1)\right]}\right]=-q'(e_1),$$

or

$$\begin{aligned}\frac{\partial e_1}{\partial b_1} &= -\frac{q'(e_1)}{q''(e_1)\left[(a_1+b_1)+sq(e_2)\right]-\frac{s^2[q'(e_2)]^2[q'(e_1)]^2}{q''(e_2)[(a_2+b_2)+sq(e_1)]}} \\ &= -\frac{q'(e_1)q''(e_2)\left[(a_2+b_2)+sq(e_1)\right]}{q''(e_1)\left[(a_1+b_1)+sq(e_2)\right]q''(e_2)\left[(a_2+b_2)+sq(e_1)\right]-s^2[q'(e_2)]^2[q'(e_1)]^2} \quad \text{(A.5)}\end{aligned}$$

It follows from (A.5) that the equilibrium effort of player 1 is increasing in his self-confidence bias as long as

$$q''(e_1)\left[(a_1+b_1)+sq(e_2)\right]q''(e_2)\left[(a_2+b_2)+sq(e_1)\right]>s^2[q'(e_2)]^2[q'(e_1)]^2. \quad \text{(A.6)}$$

From the first-order conditions we have

$$\begin{aligned}\left[(a_1+b_1)+sq(e_2)\right] &= \frac{c}{wq'(e_1)} \\ \left[(a_2+b_2)+sq(e_1)\right] &= \frac{c}{wq'(e_2)}\end{aligned}$$

Substituting these two equations into (A.6) we obtain

$$q''(e_1)\frac{c}{wq'(e_1)}q''(e_2)\frac{c}{wq'(e_2)}>s^2[q'(e_2)]^2[q'(e_1)]^2,$$

or

$$\frac{c^2}{w^2}\frac{q''(e_1)q''(e_2)}{q'(e_1)q'(e_2)}>s^2[q'(e_2)]^2[q'(e_1)]^2,$$

or

$$c^2q''(e_1)q''(e_2)>s^2w^2[q'(e_2)]^3[q'(e_1)]^3.$$

Making use (A.2) and (A.3) we have

$$c^2(1-\gamma)^2\beta^2\gamma^2e_1^{\gamma-2}e_2^{\gamma-2}>s^2w^2\beta^6\gamma^6e_1^{3\gamma-3}e_2^{3\gamma-3},$$

or

$$c^2(1-\gamma)^2e_1^{1-2\gamma}e_2^{1-2\gamma}>s^2w^2\beta^4\gamma^4. \quad \text{(A.7)}$$

If equilibrium effort is greater than 1 and $\gamma \in (0, 1/2]$, then inequality (A.7) is satisfied provided that

$$c^2(1-\gamma)^2 > s^2 w^2 \beta^4 \gamma^4. \tag{A.8}$$

Hence, if (A.8) holds, then the equilibrium effort of player 1 is increasing in his self-confidence bias, that is,

$$\frac{\partial e_1}{\partial b_1} > 0.$$

Furthermore, the equilibrium effort of player 2 is increasing with player 1's self-confidence bias since $\partial e_1/\partial b_1 > 0$, (A.4), and $q''(e_2) < 0$ imply

$$\frac{\partial e_2}{\partial b_1} > 0.$$

B Theory Comparison to Gervais and Goldstein (2007)

This Appendix summarizes the theory model by Gervais and Goldstein (2007) and its predictions.[1] It also shows that our theory model makes similar predictions. Gervais and Goldstein (2007) assume that production derives from a single one-period project, which can either succeed or fail with probability π and $1-\pi$, respectively. The project generates $\sigma > 0$ dollars if it succeeds, and zero if it fails. Therefore, the firm's expected profit is given by $\pi\sigma$. The probability of success π depends on the choice of effort $e_i \in [0,1]$ by each player i and is given by

$$\pi = a_1 e_1 + a_2 e_2 + s e_1 e_2,$$

where $a_i \geq 0$, $s > 0$, and $a_1 + a_2 + s < 1$. The parameter a_i is interpreted as the ability of player i. The parameter s captures the effect of the interaction between the two players. Since $s > 0$ the two players create positive externalities on each other. The players are risk neutral, choose their effort to maximize their expected utility, and sustain a private utility cost of effort given by $c(e_i) = e_i^2/2$. Player 1 is unbiased or rational whereas player 2 suffers from a self-confidence bias. Moreover, player 2 thinks that he is more skilled than he really is, and therefore overestimates the contribution of his effort to the project's chance of success. Specifically, he thinks his ability is $\tilde{a}_2 \geq a_2$, although it is actually only a_2. Hence, player 2's self-confidence bias is $b \equiv \tilde{a}_2 - a_2 \in [0, 1 - a_1 - a_2 - s)$. Player i is paid w_i if the project succeeds, and zero if it fails. Hence, player 2 solves the following maximization problem

$$\max_{e_2 \in [0,1]} w_2[a_1 e_1 + (a_2 + b)e_2 + s e_1 e_2] - \frac{1}{2} e_2^2.$$

From this it follows that player 2 chooses

$$e_2 = w_2(a_2 + b + s e_1).$$

A similar maximization problem for the rational player 1 gives

$$e_1 = w_1(a_1 + s e_2).$$

Lemma 1 in Gervais and Goldstein (2007) shows that the equilibrium efforts are given by

$$e_1 = \frac{[a_1 + (a_2 + b) s w_2] w_1}{1 - s^2 w_1 w_2},$$

[1] We do not explain the firm's choice of compensation contracts since this is neither part of our theory model or our experiment.

and

$$e_2 = \frac{(a_2 + b + a_1 s w_1) w_2}{1 - s^2 w_1 w_2}.$$

It is easy to verify that the equilibrium effort levels of the two players are increasing in w_1, w_2, a_1, a_2, s, and b. Next, Proposition 1 in Gervais and Goldstein (2007) shows that (i) firm value, $F = (\sigma - w_1 - w_2)(a_1 e_1 + a_2 e_2 + s e_1 e_2)$, is increasing in b; (ii) the payoff of player 1 is increasing in b; and (iii) the payoff of player 2 is increasing in b if and only if

$$b < \frac{s(a_1 + a_2 s w_2) w_1}{1 - 2s^2 w_1 w_2},$$

that is, if his overconfidence is not too extreme. Note that in the special case where the entire profit is distributed and split equally between the two players we have

$$w_1 = w_2 = \frac{\sigma}{2}.$$

As we have seen, our theory model assumes team revenue is deterministic and given by

$$R = 2w[a_1 q(e_1) + a_2 q(e_2) + s q(e_1) q(e_2)],$$

where $a_i > 0$, $s > 0$, and $q(e_i)$ satisfies $q'(e_i) > 0$ and $q''(e_i) < 0$. The players are risk neutral, choose their effort to maximize their perceived utility, and sustain a private utility cost of effort given by $c(e_i) = c e_i$, with $c > 0$. Both players can be biased. Player i's self-confidence bias is $b_i \equiv \tilde{a}_i - a_i$. Player i receives half of team revenue and hence solves the following maximization problem

$$\max_{e_i} w[(a_i + b_i) q(e_i) + a_j q(e_j) + s q(e_i) q(e_j)] - c e_i.$$

Hence, the equilibrium effort levels of players 1 and 2 satisfy

$$w\left[(a_1 + b_1) + s q(e_2)\right] q'(e_1) = c,$$

and

$$w\left[(a_2 + b_2) + s q(e_1)\right] q'(e_2) = c.$$

Since $q(e_i)$ is strictly concave and $c(e_i)$ is linear, the second-order conditions are satisfied. Furthermore, as we have seen in Appendix A, assuming

$$q(e_i) = \alpha + \beta e_i^{\gamma},$$

with $\alpha, \beta > 0$, $\gamma \in (0, 1/2]$, and $c^2(1-\gamma)^2 > s^2 w^2 \beta^4 \gamma^4$ implies the equilibrium effort of player i is increasing in w, a_1, a_2, s, b_i and b_j.

C Derivation of Hypotheses 4, 5, and 6

This appendix shows that our theory model implies: (i) the payoff of player i is increasing in b_j; (ii) the payoff of player i is inversely u-shaped in his self-confidence bias b_i; (iii) an increase in a player i's self-confidence bias raises team payoff. The equilibrium payoff of player i is

$$U_i = w[a_i q(e_i) + a_j q(e_j) + s q(e_i) q(e_j)] - c e_i,$$

where e_i and e_j are the equilibrium efforts of the two players given by the first-order conditions in Appendix A. We know from Appendix A that an increase in player j's self-confidence bias b_j raises the equilibrium effort of player j, e_j. Since the payoff of player i increases in the effort of player j, an increase in b_j raises the payoff of player i. This proves (i). The effect of an increase in b_i on U_i is given by

$$\begin{aligned}
\frac{\partial U_i}{\partial b_i} &= w\left[a_i q'(e_i)\frac{\partial e_i}{\partial b_i} + a_j q'(e_j)\frac{\partial e_j}{\partial b_i} + s q'(e_i) q(e_j)\frac{\partial e_i}{\partial b_i} + s q(e_i) q'(e_j)\frac{\partial e_j}{\partial b_i}\right] - c\frac{\partial e_i}{\partial b_i} \\
&= \left[w q'(e_i)\left[a_i + s q(e_j)\right] - c\right]\frac{\partial e_i}{\partial b_i} + w q'(e_j)\left[a_j + s q(e_i)\right]\frac{\partial e_j}{\partial b_i} \\
&= \left[w q'(e_i)\left[a_i + s q(e_j)\right] - c\right]\frac{\partial e_i}{\partial b_i} + w q'(e_j)\left[a_j + s q(e_i)\right]\frac{\partial e_j}{\partial b_i} \\
&= \left[w q'(e_i)\left[a_i + b_i - b_i + s q(e_j)\right] - c\right]\frac{\partial e_i}{\partial b_i} + w q'(e_j)\left[a_j + s q(e_i)\right]\frac{\partial e_j}{\partial b_i} \\
&= \left[w q'(e_i)\left[a_i + b_i + s q(e_j)\right] - c\right]\frac{\partial e_i}{\partial b_i} - b_i w q'(e_i)\frac{\partial e_i}{\partial b_i} + w q'(e_j)\left[a_j + s q(e_i)\right]\frac{\partial e_j}{\partial b_i} \\
&= -b_i w q'(e_i)\frac{\partial e_i}{\partial b_i} + w q'(e_j)\left[a_j + s q(e_i)\right]\frac{\partial e_j}{\partial b_i} \\
&= -b_i w q'(e_i)\frac{\partial e_i}{\partial b_i} - \frac{s w\left[a_j + s q(e_i)\right]\left[q'(e_j)\right]^2 q'(e_i)}{q''(e_j)\left[a_j + b_j + s q(e_i)\right]}\frac{\partial e_i}{\partial b_i} \\
&= w q'(e_i)\left[-b_i - \frac{s\left[a_j + s q(e_i)\right]\left[q'(e_j)\right]^2}{q''(e_j)\left[a_j + b_j + s q(e_i)\right]}\right]\frac{\partial e_i}{\partial b_i}.
\end{aligned}$$

We know from Appendix A that $\partial e_i/\partial b_i > 0$. Hence, $\partial U_i/\partial b_i > 0$ when

$$-b_i - \frac{s\left[a_j + s q(e_i)\right]\left[q'(e_j)\right]^2}{q''(e_j)\left[a_j + b_j + s q(e_i)\right]} > 0,$$

or

$$b_i < -s\frac{\left[q'(e_j)\right]^2}{q''(e_j)}\frac{a_j + s q(e_i)}{a_j + b_j + s q(e_i)}.$$

Since $q'(e_j) = \gamma\beta e_j^{\gamma-1}$ and $q''(e_j) = -(1-\gamma)\gamma\beta e_j^{\gamma-2}$ we have

$$b_i < s\frac{\gamma^2\beta^2 e_j^{2(\gamma-1)}}{(1-\gamma)\gamma\beta e_j^{\gamma-2}}\frac{a_j + s q(e_i)}{a_j + b_j + s q(e_i)},$$

or

$$b_i < s \frac{\gamma \beta e_j^{\gamma}}{1-\gamma} \frac{a_j + s(\alpha + \beta e_i^{\gamma})}{a_j + b_j + s(\alpha + \beta e_i^{\gamma})}. \tag{A.9}$$

This inequality indicates that the payoff of player i is increasing in his self-confidence bias b_i for low values of the bias and decreasing with his self-confidence bias for high values of the bias (an inversely u-shaped relationship). However, since the equilibrium efforts e_i and e_j are themselves a function of b_i we cannot know for sure this relationship holds. However, we can show that this relationship holds in our theory model presented in the paper, that is, assuming $\alpha = 0$, $\beta = 1$, $\gamma = 1/2$. Under this specification we have

$$e_i = k \left[w(a_i + b_i) + \frac{sw^2}{2c}(a_j + b_j) \right]^2,$$

where $k = (2c)^2/(4c^2 - s^2 w^2)^2$. Hence, (A.9) becomes

$$b_i < s\sqrt{k} \left[w(a_j + b_j) + \frac{sw^2}{2c}(a_i + b_i) \right] \frac{a_j + s\sqrt{k}\left[w(a_i + b_i) + \frac{sw^2}{2c}(a_j + b_j) \right]}{a_j + b_j + s\sqrt{k}\left[w(a_i + b_i) + \frac{sw^2}{2c}(a_j + b_j) \right]}.$$

Taking $i = 2$ and $j = 1$ we have

$$b_2 < s\sqrt{k} \left[w(a_1 + b_1) + \frac{sw^2}{2c}(a_2 + b_2) \right] \frac{a_1 + s\sqrt{k}\left[w(a_2 + b_2) + \frac{sw^2}{2c}(a_1 + b_1) \right]}{a_1 + b_1 + s\sqrt{k}\left[w(a_2 + b_2) + \frac{sw^2}{2c}(a_1 + b_1) \right]}. \tag{A.10}$$

Let us now assume $b_1 = 0$ and $b_2 > 0$. In this case (A.10) becomes

$$b_2 < s\sqrt{k} \left[wa_1 + \frac{sw^2}{2c}(a_2 + b_2) \right],$$

or

$$b_2 < s \frac{2c}{4c^2 - s^2 w^2} \left[wa_1 + \frac{sw^2}{2c}(a_2 + b_2) \right],$$

or

$$\left(4c^2 - s^2 w^2\right) b_2 < s \left[2wca_1 + sw^2(a_2 + b_2) \right],$$

or

$$\left(4c^2 - 2s^2 w^2\right) b_2 < sw \left(2ca_1 + swa_2\right),$$

or

$$b_2 < \frac{sw\left(2ca_1 + swa_2\right)}{4c^2 - 2s^2 w^2}$$

This proves (ii) when $\alpha = 0$, $\beta = 1$, $\gamma = 1/2$, $b_1 = 0$, and $b_2 = b$. Note further that we can collapse our model to Gervais and Goldstein (2007) by assuming $b_1 = 0$, $b_2 = b$, $c = 1/2$, $w_1 = w$, and $w_2 = w$. Under this specification (A.10) becomes

$$b < sw_1\sqrt{k}\left[a_1 + sw_2(a_2 + b) \right],$$

or

$$b < sw_1 \frac{1}{1 - s^2 w_1 w_2} \left[a_1 + sw_2(a_2 + b) \right],$$

or

$$b < \frac{sw_1}{1 - s^2 w_1 w_2} \left[a_1 + sw_2(a_2 + b) \right],$$

or

$$(1 - s^2 w_1 w_2) b < sw_1 a_1 + s^2 w_1 w_2 a_2 + s^2 w_1 w_2 b,$$

or

$$(1 - 2s^2 w_1 w_2) b < sw_1 a_1 + s^2 w_1 w_2 a_2,$$

or

$$b < \frac{s(a_1 + a_2 s w_2) w_1}{1 - 2s^2 w_1 w_2}.$$

This last inequality is identical to that provided by Gervais and Goldstein (2007) in Proposition 1 (see Appendix B).

Team payoff is given by

$$\pi = 2w[a_i q(e_i) + a_j q(e_j) + sq(e_i)q(e_j)] - c(e_i + e_j).$$

The effect of an increase in player i's bias on π is given by

$$\begin{aligned}
\frac{\partial \pi}{\partial b_i} &= 2w \left[a_i q'(e_i) \frac{\partial e_i}{\partial b_i} + a_j q'(e_j) \frac{\partial e_j}{\partial b_i} + sq'(e_i) q(e_j) \frac{\partial e_i}{\partial b_i} + sq(e_i) q'(e_j) \frac{\partial e_j}{\partial b_i} \right] \\
&\quad - c \left(\frac{\partial e_i}{\partial b_i} + \frac{\partial e_j}{\partial b_i} \right) \\
&= \left[2wq'(e_i) \left[a_i + sq(e_j) \right] - c \right] \frac{\partial e_i}{\partial b_i} + \left[2wq'(e_j) \left[a_j + sq(e_i) \right] - c \right] \frac{\partial e_j}{\partial b_i} \\
&= \left[wq'(e_i) \left[a_i - b_i + sq(e_j) \right] + wq'(e_i) \left[(a_i + b_i) + sq(e_j) \right] - c \right] \frac{\partial e_i}{\partial b_i} \\
&\quad + \left[wq'(e_j) \left[a_j - b_j + sq(e_i) \right] + wq'(e_j) \left[(a_j + b_j) + sq(e_i) \right] - c \right] \frac{\partial e_j}{\partial b_i} \\
&= wq'(e_i) \left[a_i - b_i + sq(e_j) \right] \frac{\partial e_i}{\partial b_i} + wq'(e_j) \left[a_j - b_j + sq(e_i) \right] \frac{\partial e_j}{\partial b_i}, \qquad \text{(A.11)}
\end{aligned}$$

where the last equality follows from the first-order conditions of players i and j. Recall from Appendix A that $\partial e_i / \partial b_i > 0$ and $\partial e_j / \partial b_i > 0$. Hence, it follows from (A.11) that if a team is composed of two underconfident players, i.e., $b_i < 0$ and $b_j < 0$, then $\partial \pi / \partial b_i > 0$. It also follows from (A.11) that if a team is composed of one overconfident player i and one underconfident player j, i.e., $b_i > 0$ and $b_j < 0$, then a sufficient condition for $\partial \pi / \partial b_i > 0$ is that $a_i - b_i > 0$. Furthermore, it follows from (A.11) that if a team is composed of two

overconfident players, i.e., $b_i > 0$ and $b_j > 0$, then a sufficient condition for $\partial\pi/\partial b_i > 0$ is that $a_i - b_i > 0$ and $a_j - b_j > 0$. In other words, in a team composed of two overconfident players, a sufficient condition for an increase in player i's overconfidence to raise team payoff is that each player's overconfidence bias is less than that player's true ability. Note also that if a team is composed of two overconfident players who have a true ability above the median, then $b_i < a_i$ and $b_j < a_j$, and therefore $\partial\pi/\partial b_i > 0$. This means that if a team is composed of two overconfident players, the sufficient condition only matters when at least one player has a true ability below the median. The intuition behind this result is that a very large overconfidence bias can lead the players to choose effort levels above the first-best. When that happens, an increase in player i's overconfidence lowers team payoff.

Note that the first-best effort levels are the solution to

$$\max_{e_i,e_j} 2w[a_i q(e_i) + a_j q(e_j) + sq(e_i)q(e_j)] - c(e_i + e_j).$$

The first-order conditions of this problem are

$$\begin{aligned} \frac{\partial R(e_i,e_j)}{\partial e_i} - c &= 2w[a_i + sq(e_j)]q'(e_i) - c = 0 \\ \frac{\partial R(e_i,e_j)}{\partial e_j} - c &= 2w[a_j + sq(e_i)]q'(e_j) - c = 0 \end{aligned} \tag{A.12}$$

When both players are unbiased, i.e., $b_i = b_j = 0$, the equilibrium effort levels are given by the first-order conditions

$$\begin{aligned} \frac{1}{2}\frac{\partial R(e_i,e_j)}{\partial e_i} - c &= w[a_i + sq(e_j)]q'(e_i) - c = 0 \\ \frac{1}{2}\frac{\partial R(e_i,e_j)}{\partial e_j} - c &= w[a_j + sq(e_i)]q'(e_j) - c = 0 \end{aligned} \tag{A.13}$$

Note that the assumption that $q(e_i)$ is concave implies that team revenue is a concave function of effort since

$$\frac{\partial^2 R(e_i,e_j)}{\partial e_i^2} = 2w[a_i + sq(e_j)]q''(e_i) < 0.$$

It follows from (A.12), (A.13), and concavity of team revenue, that when both players are unbiased, they under provide effort relative to the first-best. This is due to the free-riding problem: when a player raises his effort this increases team revenue but he only receives half of that increase. Hence, an increase in overconfidence of either player will raise the effort of both players and team payoff as long as players' efforts are below the first-best.

D Distribution of Individual Characteristics across Treatments

Table 1: Individual Characteristics across Treatments

	ALL	EASY	HARD	p-value EASY-HARD
age	21.4292	21.3917	21.4667	0.8829
	(3.9329)	(3.5911)	(4.2623)	
male	0.5375	0.6083	0.4667	0.0278
	(0.4996)	(0.4902)	(0.5010)	
risk aversion	-7.7667	-10.0417	-5.4917	0.0604
	(18.7801)	(19.4255)	(17.9040)	
preferences efficiency	0.6583	0.6583	0.6583	1
	(0.4753)	(0.4763)	(0.4763)	
inequality aversion	0.2	0.15	0.25	0.0531
	(0.4008)	(0.3586)	(0.4348)	
inequality loving	0.0333	0.0417	0.025	0.4741
	(0.1799)	(0.2007)	(0.1568)	
spiteful	0.0125	0.025	n.a.[a]	n.a.[a]
	(0.1113)	(0.1568)		
swiss	0.575	0.575	0.575	1
	(0.4954)	(0.4964)	(0.4964)	
only child	0.0708	0.0667	0.75	0.8023
	(0.2571)	(0.2505)	(0.2645)	
education parents	0.4375	0.425	0.45	0.6977
	(0.4971)	(0.4964)	(0.4996)	
people known	0.3167	0.3333	0.3	0.6813
	(0.6269)	(0.5397)	(0.7053)	
big town	0.5542	0.5583	0.55	0.8972
	(0.4981)	(0.4987)	(0.4996)	
unil	0.6375	0.5917	0.6833	0.1408
	(0.4817)	(0.4936)	(0.4671)	
bachelor	0.7792	0.8167	0.7417	0.1627
	(0.4157)	(0.3886)	(0.4396)	
grade	4.6	4.6583	4.65417	0.0941
	(0.5396)	(0.4936)	(0.5783)	
No. of observations	240	120	120	

Standard errors are shown in parentheses.

[a] The difference on spiteful could not be estimated, as there are no spiteful subjects in the HARD treatment.

E Effect of Ability Multiplier on Effort Provision in Trial Periods

Figure 1: Average number of clicks per multiplier during trial periods

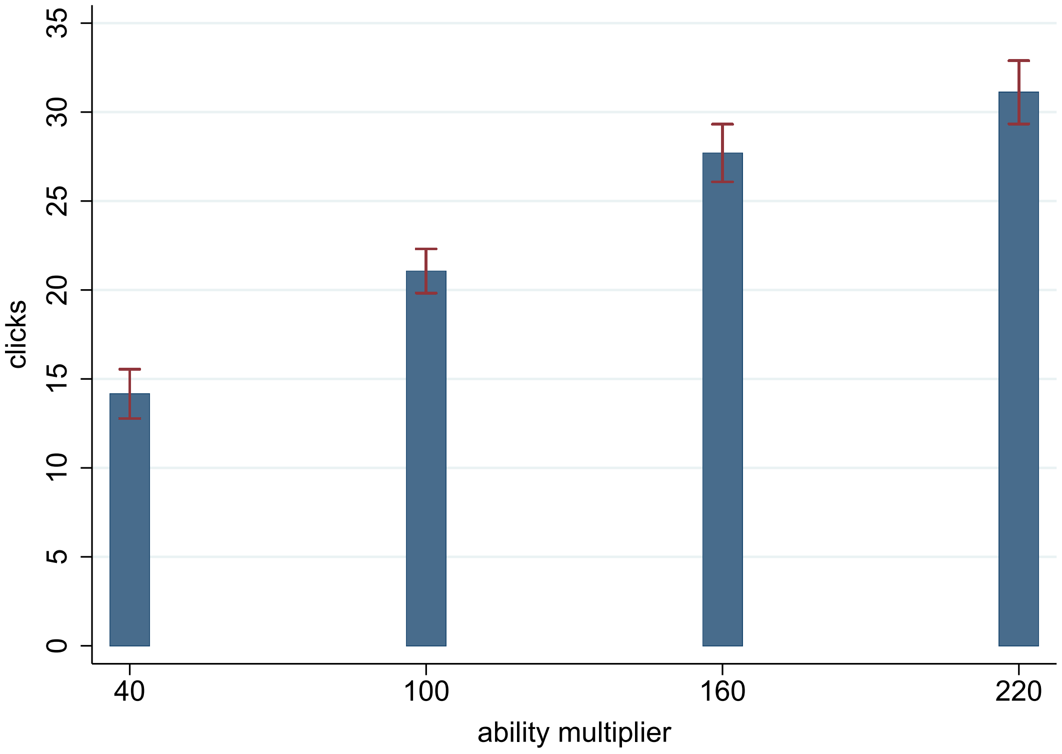

This figure depicts the average number of clicks in the trial periods for each of the four ability multipliers (40,100,160,220). The mean number of clicks is 14.16, 21.07, 27.70, and 31.11. Confidence intervals are depicted in red.

Figure 2: Density of number of clicks per multiplier during trial periods

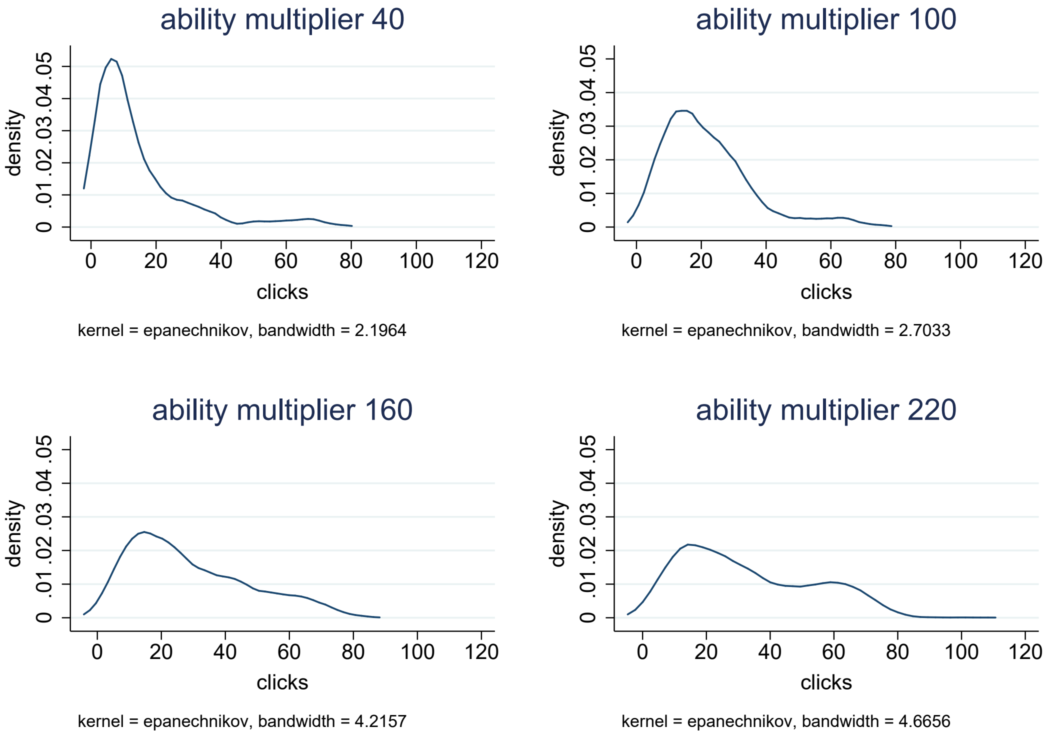

This figure displays the density of the number of clicks for each of the four multiplier.

F Adjusting for Multiple Hypotheses Testing

Since in Section 4.2 we have four outcomes on which we regress our binary treatment variable, the resulting p-values should be adjusted for multiple hypothesis testing (see for example List et al. (2019)). To the best of our knowledge the procedure proposed by List et al. (2019), with its associated *mhtexp* stata command, works only for OLS specifications without clustering. The underlying multiple hypothesis procedure used in List et al. (2019) is the Romano-Wolf test statistic (Romano and Wolf, 2005a,b, 2016). However, we employ another resample-based multiple testing statistic from Westfall and Young (1993). The Westfall-Young test statistic has the advantage to allow for clustering but, unfortunately, not for the GLS specification. Given that the clustered standard errors in OLS and in GLS with random effects are similiar, as it can be seen in Table 2, we use the Westfall-Young (WY) test statistics for the correction of multiple hypotheses. We see that the main result, concerning the number of clicks, remains significant. Resample-based test statistics are usually more efficient than simple correction procedures, such as Bonferroni or Holm, which we also report in Table 2 and which give qualitatively the same results.

Table 2: Adjusting for Multiple Hypotheses Testing

		p values				
		unadjusted		adjusted		
Regression Type		GLS	OLS	OLS		
Standard Errors		Clustered	Clustered	Clustered		
Outcome	Diff. in means			WY	Holm	Bonferroni
Clicks	4.2573	0.0201**	0.0218**	0.0489**	0.0872*	0.0872*
Catches	1.5646	0.1021	0.1048	0.1702	0.3143	0.4190
Team revenue	3.0045	0.3417	0.3437	0.3741	0.6873	1
Individual payoffs	0.7927	0.5663	0.5673	0.5689	0.5673	1

WY stands for Westfall-Young. Significantly different from zero at 1% (***), 5% (**), 10% (*).

G Distribution of Clicks and Catches per Treatment

Figure 3: Distribution of Number of Clicks per Treatment

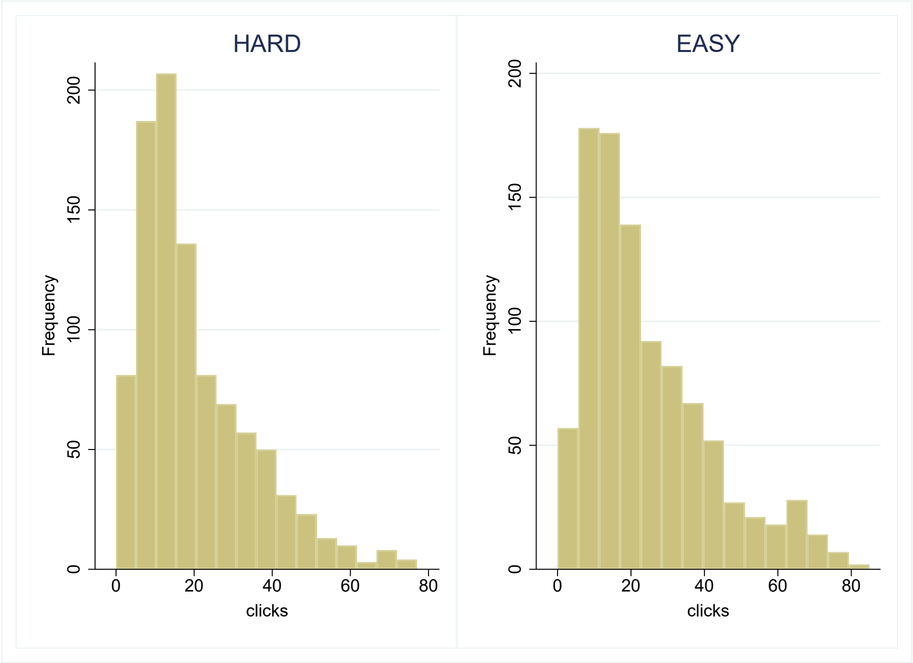

This figure displays the distribution of the number of clicks in each treatment.

Figure 4: Distribution of Number of Catches per Treatment

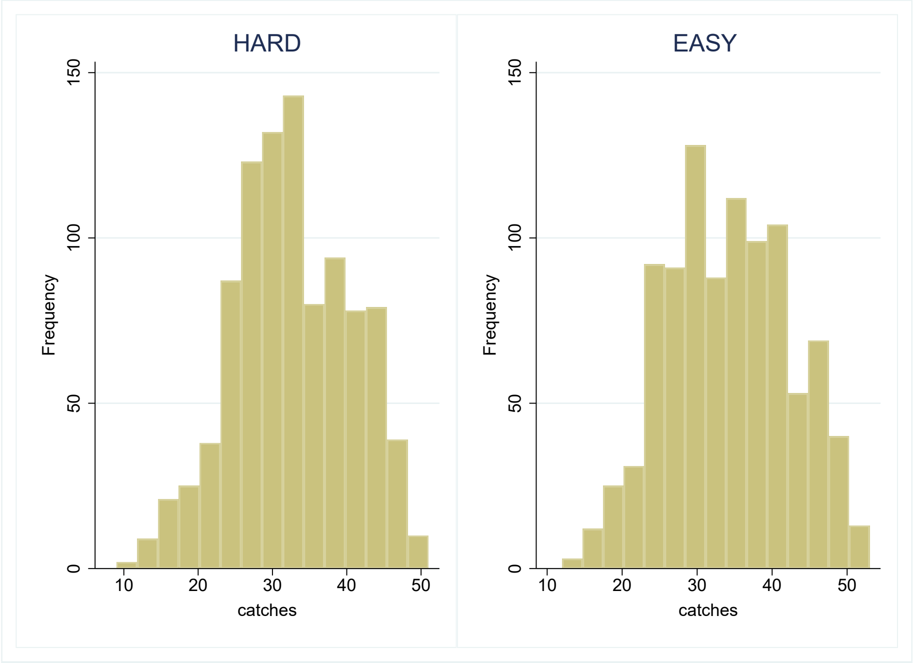

This figure displays the distribution of the number of catches in each treatment.

H Regression (4) with the Square Root of Effort as the Dependent Variable

Table 3: Square Root of Effort Regression

		Effort in squared root of clicks ($\sqrt{e_{it}}$)
constant	$\hat{\beta}_0$	2.1478**<br>(0.9235)
i's ability multiplier (a_i)	$\hat{\beta}_1$	0.0080***<br>(0.0021)
i's self-confidence bias (b_i)	$\hat{\beta}_2$	0.0093***<br>(0.0021)
j's ability multiplier (a_j)	$\hat{\beta}_3$	0.0035**<br>(0.0018)
j's self-confidence bias: (b_j)	$\hat{\beta}_4$	-0.0003<br>(0.0018)
age		0.0053<br>(0.0216)
male		0.8881***<br>(0.1774)
risk aversion		0.0009<br>(0.0046)
preferences efficiency		0.5717*<br>(0.2871)
inequality aversion		0.1792<br>(0.3210)
inequality loving		0.0061<br>(0.5946)
spiteful		1.8959<br>(1.6859)
swiss		0.0344<br>(0.1764)
only child		-0.6029***<br>(0.2690)
education parents		-0.2721<br>(0.1774)
people known		-0.0328<br>(0.1211)
big town		-0.0599<br>(0.1713)
unil		-0.1359<br>(0.2019)
bachelor		-0.3886*<br>(0.2321)
grade		0.0610<br>(0.1508)
period 2		0.0210<br>(0.0502)
period 3		-0.0236<br>(0.0640)
period 4		-0.0122<br>(0.0724)
period 5		-0.0378<br>(0.0761)
period 6		-0.0380<br>(0.0749)
period 7		0.0489<br>(0.0873)
period 8		-0.0137<br>(0.0776)
No. of observations		1,920
R^2		0.2615

The table shows the results of Regression (4) with all controls with the dependant being the squared root of the number of clicks instead of the number of clicks. These are GLS regressions with random effects and standard errors clustered at the team level. Significantly different from zero at 1% (***), 5% (**), 10% (*).

I Regression Results Showing all Controls

Table 4: Effort and Team Revenue Regressions Showing all Controls

	Effort in Clicks (e_{it})		Team Revenue in CHF (R_{ijt})	
constant	$\hat{\beta}_0$	1.1244<br>(8.6476)	$\hat{\gamma}_0$	5.8524<br>(15.0389)
i's ability multiplier (a_i)	$\hat{\beta}_1$	0.0718***<br>(0.0213)	$\hat{\gamma}_1$	0.1722***<br>(0.0266)
i's self-confidence bias (b_i)	$\hat{\beta}_2$	0.0871<br>(0.0213)	$\hat{\gamma}_2$	0.0300<br>(0.0201)
j's ability multiplier (a_j)	$\hat{\beta}_3$	0.0396**<br>(0.0176)	$\hat{\gamma}_3$	0.1772***<br>(0.0210)
j's self-confidence bias: (b_j)	$\hat{\beta}_4$	0.0028<br>(0.0181)	$\hat{\gamma}_4$	0.0521**<br>(0.0236)
age		0.1263<br>(0.2114)		-0.2661<br>(0.3860)
male		8.1427***<br>(1.6608)		
male-male				9.2785***<br>(2.0941)
male-female				3.0305*<br>(1.8035)
risk aversion		0.0009<br>(0.0430)		0.0025<br>(0.0522)
preferences efficiency		5.2404*<br>(2.6902)		6.7502<br>(4.3426)
inequality aversion		1.3932<br>(2.9021)		3.0349<br>(4.3050)
inequality loving		0.9307<br>(6.1875)		-1.6793<br>(9.7068)
spiteful		23.3585<br>(17.5933)		3.2757<br>(14.7489)
swiss		-0.0918<br>(1.6478)		
swiss-swiss				-.4619<br>(2.4423)
swiss-other				2.0626<br>(2.2273)
only child		-5.9575**<br>(2.3888)		2.7887<br>(2.9930)
education parents		-2.8587<br>(1.7355)		-3.2165<br>(2.8080)
people known		-0.6506<br>(1.1890)		1.1922<br>(2.1965)
big town		-1.3328<br>(1.7030)		0.2069<br>(2.1676)
unil		-1.1860<br>(2.0221)		1.6803<br>(3.2660)
bachelor		-3.9396*<br>(2.2139)		-9.1593***<br>(3.3624)
grade		0.2492<br>(1.5035)		0.2566<br>(2.4192)
period 2		0.3667<br>(0.4988)		-0.1335<br>(0.6862)
period 3		0.0458<br>(0.6471)		-0.3228<br>(0.7495)
period 4		0.1708<br>(0.7255)		-0.8714<br>(0.8413)
period 5		0.025<br>(0.7459)		-0.7154<br>(0.9278)
period 6		0.05<br>(0.7383)		-2.0349**<br>(0.8791)
period 7		0.9375<br>(0.9149)		-1.3835<br>(0.9348)
period 8		0.125<br>(0.7690)		-1.1808<br>(0.8913)
No. of observations		1,920		960
R^2		0.2497		0.7139

The table shows the results of Regressions (4) and (5) with all controls. In the regression on team revenue, controls are averaged over both teammates, except for the gender and nationality composition of the team and the period dummies. These are GLS regressions with random effects and standard errors clustered at the team level. Significantly different from zero at 1% (***), 5% (**), 10% (*).

Table 5: Payoff Regressions Showing all Controls

	Payoff in CHF (U_{it})		Team Payoff in CHF (π_{ijt})	
constant	$\hat{\delta}_0$	3.3487<br>(3.3344)	$\hat{\eta}_0$	2.3941<br>(11.2861)
i's ability multiplier (a_i)	$\hat{\delta}_1$	0.0747***<br>(0.0061)	$\hat{\eta}_1$	0.1497***<br>(0.0199)
i's self-confidence bias (b_i)	$\hat{\delta}_2$	0.0089<br>(0.0061)	$\hat{\eta}_2$	0.0143<br>(0.0164)
i's self-confidence bias squared (b_i^2)	$\hat{\delta}_3$	-0.0001<br>(0.0001)		
j's ability multiplier (a_j)	$\hat{\delta}_4$	0.0870***<br>(0.0061)	$\hat{\eta}_3$	0.1587***<br>(0.0143)
j's self-confidence bias: (b_j)	$\hat{\delta}_5$	0.0225***<br>(0.0061)	$\hat{\eta}_4$	0.0346**<br>(0.0170)
age		-0.0907<br>(0.0948)		-0.2656<br>(0.2984)
male		0.9861*<br>(0.5357)		
male-male				6.5311***<br>(1.5289)
male-female				2.3126*<br>(1.3261)
risk aversion		0.0042<br>(0.0110)		0.0141<br>(0.0390)
preferences efficiency		0.4111<br>(3.2341)		5.6406*<br>(3.2143)
inequality aversion		0.1254<br>(1.1307)		3.1371<br>(3.1300)
inequality loving		-0.5840<br>(1.6498)		-0.6175<br>(6.5961)
spiteful		-4.1905<br>(2.9916)		-1.3920<br>(10.9719)
swiss		-0.5323<br>(0.5589)		
swiss-swiss				-0.1622<br>(1.8353)
swiss-other				2.1723<br>(1.7075)
only child		-0.5105<br>(0.8256)		-1.5957<br>(2.1869)
education parents		0.1476<br>(0.5432)		-1.8537<br>(2.0460)
people known		0.7000<br>(0.3864)		0.9468*<br>(1.6170)
big town		-0.0607<br>(0.4752)		0.7176<br>(1.6278)
unil		0.4798<br>(0.6063)		1.7031<br>(2.5074)
bachelor		-1.5748**<br>(0.7687)		-6.4543***<br>(2.3537)
grade		-0.2203<br>(0.5266)		0.3828<br>(1.8668)
period 2		-0.1278<br>(0.3102)		-0.2557<br>(0.6252)
period 3		-0.1690<br>(0.3349)		-0.3381<br>(0.6751)
period 4		-0.4642<br>(0.3472)		-0.9283<br>(0.6998)
period 5		-0.3619<br>(0.3983)		-0.7237<br>(0.8029)
period 6		-1.0258***<br>(0.3640)		-2.0515***<br>(0.7336)
period 7		-0.8480**<br>(0.3943)		-1.6960**<br>(0.7948)
period 8		-0.6113<br>(0.3831)		-1.2225<br>(0.7721)
p-value of Wald test for joint significance of b_i and b_i^2		0.227		
No. of observations		1,920		960
R^2		0.7005		0.7624

The table shows the results of Regressions (6) and (7) with all controls. In the regression on team payoff, controls are averaged over both teammates, except for the gender and nationality composition of the team and the period dummies. These are GLS regressions with random effects and standard errors clustered at the team level. Significantly different from zero at 1% (***), 5% (**), 10% (*).

J Additional Treatments

The additional treatments for detecting potential mood effects comprise 240 subjects, as many as in the two main treatments. These subjects went through the same experiment as described in Section 3, except that i) they were informed about their true ability multipliers before they started working on the team effort task and, hence, ii) they did not have to state their posterior beliefs. Half of them was exposed to the EASY general knowledge quiz and the other half to the HARD quiz. These two additional treatments allow us to check for two potential confounds: mood effects and strategic misreporting of beliefs.

J.1 Mood Effects

Table 6 summarizes the main results of the additional treatments. As we can see, the difference in average effort per period between the EASY and the HARD treatments is only 2 clicks which amounts to 8.5%. This difference is not statistically significant using either unadjusted or adjusted tests. In addition, the differences in average catches, team revenue, and individual payoffs are also not statistically significant. Overall, this shows that mood effects only play a minor role.

Table 6: Additional Treatments: Main Results

		p values				
		unadjusted		adjusted		
Regression Type		GLS	OLS	OLS		
Standard Errors		Clustered	Clustered	Clustered		
Outcome	Diff. in means			WY	Holm	Bonferroni
Catches	1.3938	0.2140	0.2164	0.3579	0.8657	0.8657
Clicks	2.0021	0.3975	0.3992	0.5493	1	1
Team revenue	2.2020	0.5334	0.5346	0.5708	1	1
Individual payoffs	0.7673	0.5964	0.5974	0.5919	0.5974	1

WY stands for Westfall-Young. Significantly different from zero at 1% (***), 5% (**), 10% (*).

J.2 Strategic Misreporting

We analyze whether subjects strategically misreport their beliefs in two ways. First, we compare self-confidence bias between the main treatments and the additional treatments, where there is no incentive to misreport. Second, we explore whether risk tolerant subjects overstate their beliefs more than risk averse subjects in the main treatment.

Table 7 reports the results. Column (3) shows the coefficients of a fully saturated OLS regression of the subjects' self-confidence bias on binary indicators for being exposed to the EASY quiz and being in the Additional Treatments. As the indicator for the Additional Treatments is not significant, neither in levels nor interacted with EASY, there is no evidence that subjects's self-confidence bias in the main treatments is any different from those of subjects in the additional treatments. Columns (1) and (2) show the coefficients of OLS regressions of subjects' self-confidence bias in the main treatment on elicited risk aversion, without and with controls, respectively. Since the coefficient on risk aversion is not significant, risk tolerant subjects do not overstate their beliefs more than risk averse subjects. Hence, in both approaches we find no evidence for strategic misreporting.

Table 7: Testing for Strategic Misreporting

i's self-confidence bias	(1)	(2)	(3)
constant	6.2065	28.5934	-9.6667*
	(4.7059)	(63.8849)	(5.8566)
risk aversion	-0.1236	-0.0059	
	(0.2238)	(0.2396)	
EASY			33.6667***
			(8.3171)
Additional Treatments			-6.1667
			(8.3544)
EASY × Additional Treatments			-0.1667
			(11.7561)
Treatments	Main	Main	Main & Additional
Controls	no	yes	no
No. of observations	240	240	480
R^2	0.0012	0.0902	0.0656

The first and second columns display OLS regressions with risk aversion as the main regressor, without and with controls, respectively. The third column displays a fully saturated OLS regression with indicators for being exposed to the EASY quiz and being assigned to the Additional Treatments. Standard errors are clustered at the team level. Significantly different from zero at 1% (***), 5% (**), 10% (*).

K Effort and Team Revenue Regressions Constrained on the First Period

As a robustness check we constrain the regressions on effort in clicks and team revenue, equations 4 and 5, respectively, on period 1 behavior. Table 8 summarizes the results. In line with the results obtained in the regression on effort in clicks for all periods, i's ability multiplier and self-confidence bias are significant at 1%. In contrast, j's ability multiplier is not significant when conditioning on period 1 behavior. However, as Figure 5 shows, conditioning only on period 2 and only on each of the subsequent periods, j's ability multiplier is significant in all periods, except period 5. This suggests the information disclosed to the subjects in period 1 was helpful in reinforcing their understanding that they are in a team setting and that, on average, teammates with higher ability multipliers are more productive. The regression on team revenue conditioning on period 1 behavior shows qualitatively the same results as the one pooling all periods.

Table 8: Effort and Team Revenue Regressions Constrained on First Period

		Effort in Clicks (e_{i1})			Team Revenue in CHF (R_{ij1})	
constant	$\hat{\beta}_0$	3.3088<br>(3.1136)	11.7768<br>(10.0162)	$\hat{\gamma}_0$	-3.3599<br>(4.5836)	18.9657<br>(17.4864)
i's ability multiplier (a_i)	$\hat{\beta}_1$	0.1160***<br>(0.0198)	0.0966***<br>(0.0233)	$\hat{\gamma}_1$	0.1882***<br>(0.0273)	0.1882***<br>(0.0309)
i's self-confidence bias (b_i)	$\hat{\beta}_2$	0.1270***<br>(0.0248)	0.1187***<br>(0.0265)	$\hat{\gamma}_2$	0.0700***<br>(0.0217)	0.0749***<br>(0.0238)
j's ability multiplier (a_j)	$\hat{\beta}_3$	0.0215<br>(0.0177)	0.0222<br>(0.0175)	$\hat{\gamma}_3$	0.1914***<br>(0.0232)	0.1939***<br>(0.0274)
j's self-confidence bias: (b_j)	$\hat{\beta}_4$	-0.0073<br>(0.0196)	-0.0146<br>(0.0197)	$\hat{\gamma}_4$	0.0526**<br>(0.0264)	0.0460<br>(0.0281)
Controls (X and P)		no	yes		no	yes
No. of observations		240	240		120	120
R^2		0.1653	0.2448		0.6798	0.7236

The table shows the results of Regressions (4) and (5) for the first period of the ball catching task with and without controls. These are OLS regressions with standard errors clustered at the team level. Significantly different from zero at 1% (***), 5% (**), 10% (*).

Figure 5: Partner Ability Multiplier Coefficients Plot

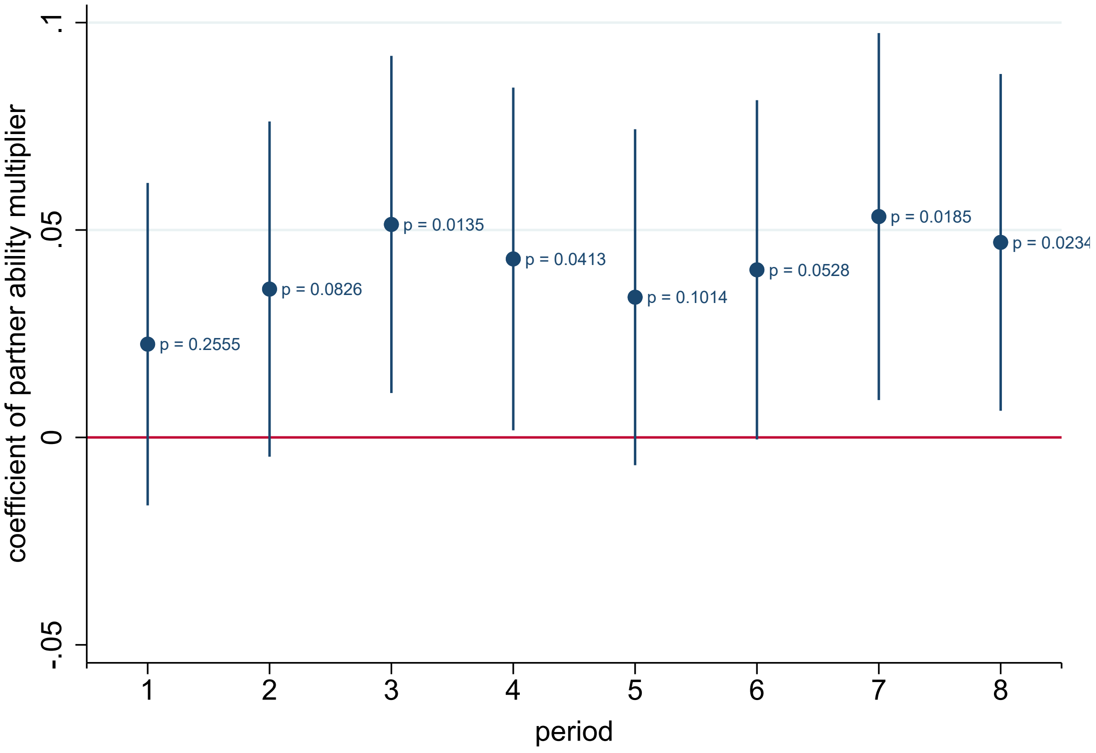

The figure displays the estimated coefficient of the Partner Ability Multiplier $\hat{\beta}_3$ for each period separately. The estimated coefficients are based on Regression (4) for each period separately. The blue lines represent 95 % confidence intervals.

L Experimental Instructions

This appendix contains an English translation of the experimental instructions and the control questions.

General Instructions (Y)

Welcome to the experiment. You are about to participate in an experiment on decision making. We thank you for participating in our economic study.

Throughout the experiment you must not communicate with other participants. If you have a question or need assistance of any kind, please raise your hand, and a study administrator will come to your seat and you can discuss the question. The violation of the rule against communication will result in exclusion from the study and from all payments.

This experiment consists of a total of **7 parts**:

- In the **first part**, we ask you to answer a general knowledge quiz.
- In the **second part**, we ask you to make an estimate.
- In the **third part**, you are paired with another participant in your group and we ask both of you to perform a ball-catching task.
- In the **fourth part**, you have the possibility of making another estimate.
- In the **fifth part**, you are paired with yet another participant in your group and we ask both of you to make distributional decisions.
- In the **sixth part**, we ask you to make one risky decision.
- In the **seventh part**, we ask you to complete a questionnaire.

After you read the general instructions, you can proceed to the detailed instructions for the 7 parts of the experiment. The amount of money you will be paid in this experiment depends on your decisions. Therefore, **it is in your interest to read the general and detailed instructions carefully.**

How are the payments in this experiment determined?

1. You receive a fixed payment of CHF 5 for participating in the experiment. Additionally, you will also receive the payments described below.
2. In the **first part**, your payment depends on your relative performance on the general knowledge quiz within your group of 12 participants. Here, your payment can go from 720 tokens to 60 tokens.
3. In the **second part**, your payment depends on the accuracy of your estimate and can be either 600 tokens or 0 tokens.
4. In the **third part**, your payment depends on your and your matched participant's performance on the general knowledge quiz and on the ball-caching task. Here, the average payment is about 5000 tokens.

5. In the **fourth part**, if you make another estimate your payment depends on the accuracy of your estimate and the realization of a random device.
6. In the **fifth part**, your payment depends on your distributional decisions, those of a matched participant, and on the realization of a random device. Here, your payment can go from 315 tokens to 185 tokens.
7. In the **sixth part**, your payment depends on your risky decision and on the realization of a random device. Here, your payment can go from 742.5 tokens to 0 tokens.

At the end of the experiment, the number of tokens you have earned will be exchanged into CHF using the exchange rate:

300 tokens = 1 CHF

Please know that **your anonymity is guaranteed**. Also, you will not be informed of the identity of the participants who are paired with you.

Overview of the experiment

**First Part:**<br>Answer a general knowledge quiz
↓
**Second Part:**<br>Make an estimate
↓
**Third Part**<br>Perform the ball-catching task
↓
**Fourth Part:**<br>Option to make another estimate
↓
**Fifth Part:**<br>Make distributional decisions
↓
**Sixth Part:**<br>Make one risky decision
↓
**Seventh Part:**<br>Complete a questionnaire

First Part:

Answer a general knowledge quiz

As you walked into this room you were randomly assigned to a group of 12 participants. You will be in this group for the entire experiment.

In this first part of the experiment you will be asked to provide answers to 46 questions of general knowledge. You have 20 minutes for this purpose. The more questions you will be able to answer correctly, the higher your earnings in this task. If you are the best among the 12 participants in your group (rank 1), you will earn 720 tokens and for each subsequent rank, the number of tokens earned decreases by 60. For example, if you are the second-best in your group (rank 2), you receive 660 tokens, if you are the third-best in your group (rank 3) you receive 600 tokens, …, if you are the worst participant in your group (rank 12), you receive 60 tokens. Ties will be broken randomly. Furthermore, **the more questions you will be able to answer correctly, the higher your earnings will be in the third part of the experiment**. Hence, it is in your interest to provide as many correct answers as possible.

Note that the correct answers to some questions imply that you give the first and last name of famous people. You will get a full credit only if you get the first and last name of the relevant famous person. If you only write the correct first name or last name, you will get half credit for your answer. Questions without answers are considered as incorrect.

Second Part:

Make an estimate

In this second part of the experiment, you will be asked to provide an estimate. This estimate is about your rank in the quiz. In the following, we will explain you how you will indicate your estimate of your rank and how this estimate influences your earnings.

a) How will you indicate your estimate of your rank?

The 12 participants in your group, including you, have answered the same quiz. According to the performance of all 12 participants in your group, each of them gets ranked. Rank 1 is assigned to the participant with the best performance in your group (in other words, this is the participant with the highest number of correct answers); rank 2 is assigned to the participant with the second best performance in your group,…, rank 12 is assigned to the participant with the worst performance in your group. We wish you to provide us with your estimate of your rank as an integer between 1 and 12.

b) How does your estimate of your rank influence your earnings?

The more your estimate of your rank is precise, the more likely it is that you will earn 600 tokens. In other words, the likelihood you earn the 600 tokens is greater the closer your estimate of your rank is to your real rank.

Your earnings in this part of the experiment are determined as follows.

First, the computer will randomly generate a number between 0 and 121. Each number from 0 to 121 is equally likely.

Second, the difference between your estimate of your rank and your real rank is your prediction error. If your prediction error multiplied by itself is not greater than the randomly generated number, you win **600 tokens**. Otherwise, you win **0 tokens**.

Important: You might wonder why we have chosen this payment rule. The reason is that this payment rule makes it optimal for you to state truthfully your estimate of your rank.

Example: Your estimate of your rank is 5, but given your relative performance at the quiz, your real rank is 8. In this case, your prediction error is 5-8 = -3. Your prediction error multiplied by itself is 9. If the randomly generated number is greater than or equal to 9, for example 35, then you win 600 tokens. If the randomly generated number is smaller than 9, for example 8, then you win 0 tokens.

Before the beginning of the third part of the experiment, you will be randomly matched to one of the other 11 participants in your group, henceforth referred to as *your partner*. In the third part of the experiment, your computer screen will display your partner's estimate of his/her rank and your partner's real rank. Similarly, your partner's computer screen will display your estimate of your rank and your real rank.

Please be aware that you will not be informed about your real rank until the very end of the experiment. Similarly, y**our partner will also not be informed about his/her real rank until the very end of the experiment.**

This is the end of the instructions of this part of the experiment. Do you have any questions? If you have questions, please raise your hand.

Third Part:

Perform the ball-catching task

In this part of the experiment, you will work on a computerized ball-catching task for **16 periods**. Each period lasts one minute. Please read these explanations carefully. You will only be able to continue the experiment after you answered correctly to control questions that will test your understanding of how your earnings in the ball-catching task are determined in each of the 16 periods.

In **periods 1 to 8**, there will be a task box in the middle of the task screen like the one shown below:

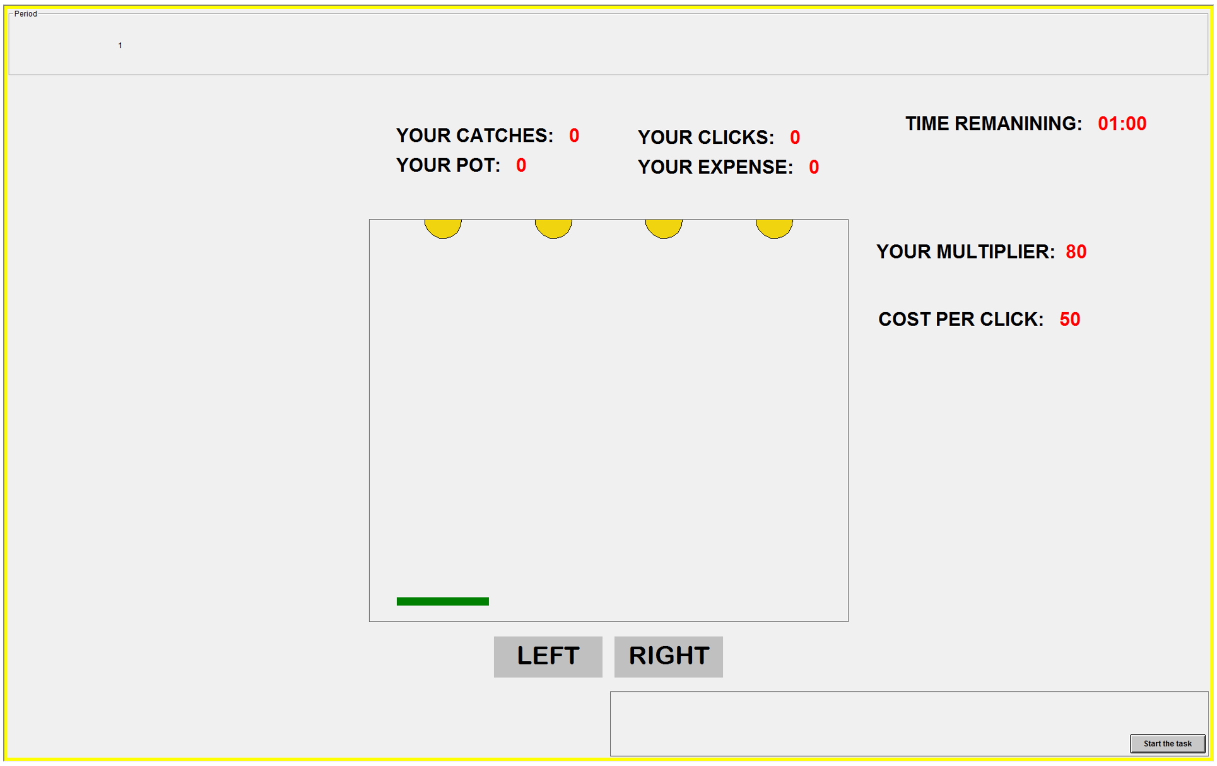

Once you click on the "Start the Task" button, the timer will start and balls will fall randomly from the top of the task box. You can move the tray at the bottom of the task box to catch the balls by using the mouse to click on the LEFT or RIGHT buttons. To catch a ball, your tray must be below the ball before it touches the bottom of the tray. When the ball touches the tray, your catches increase by one.

In **periods 1 to 8**, you will receive an amount (in tokens) for each ball you catch and incur a cost (in tokens) for each mouse click you make. At the beginning of each period you will be informed of your "multiplier". Your multiplier determines

the amount of tokens you will receive for each ball you catch and will be either 40, 100, 160, or 220 tokens. Your cost per mouse click will always be 50 tokens. Each of the four aforementioned multipliers will be valid during two consecutive periods as shown in the table below:

periods	1 and 2	3 and 4	5 and 6	7 and 8
Increasing multipliers	40	100	160	220
Decreasing multipliers	220	160	100	40

A random device will determine whether you will begin with a multiplier equal to 40 or, instead, begin with a multiplier equal to 220. Each of the two sequences of multipliers is equally likely.

In **periods 1 to 8**, the number of balls you caught so far (displayed as **YOUR CATCHES**), the number of mouse clicks you made so far (displayed as **YOUR CLICKS**), your accumulated amount of tokens so far (displayed as **YOUR POT**), and your accumulated mouse click costs so far (displayed as **YOUR EXPENSE**) are shown right above the task box. At the end of the period, **your pot** will be equal to your "multiplier" times your catches for the period and **your expense** will be equal to the cost per click of 50 tokens multiplied by your number of clicks for the period. At the end of the period, **your earnings** in tokens for the period will be **your pot** minus **your expense**. Please note that catching more balls by moving the tray more often does not necessarily lead to higher earnings because both **your pot** and **your expense** matter for determining **your earnings**.

At the end of each period from 1 to 8, you will see on your computer screen how many balls you caught, your clicks, and your earnings for that particular period.

In **periods 9 to 16** your rank in the quiz determines your multiplier as follows:

rank in quiz	Multiplier
1	240
2	220
3	200

4	180
5	160
6	140
7	120
8	100
9	80
10	60
11	40
12	20

For example, if your rank in the quiz was 5, then your multiplier is 160. However, if your rank in the quiz was 8, then your multiplier is 100. In other words, the better your performance in the quiz is, the higher is your multiplier. Note that your partner's rank in the quiz determines his/her multiplier in the same way. For example, if your partner's rank in the quiz was 6, then his/her multiplier is 140.

In **periods 9 to 16** the task box in the middle of the screen is shown below:

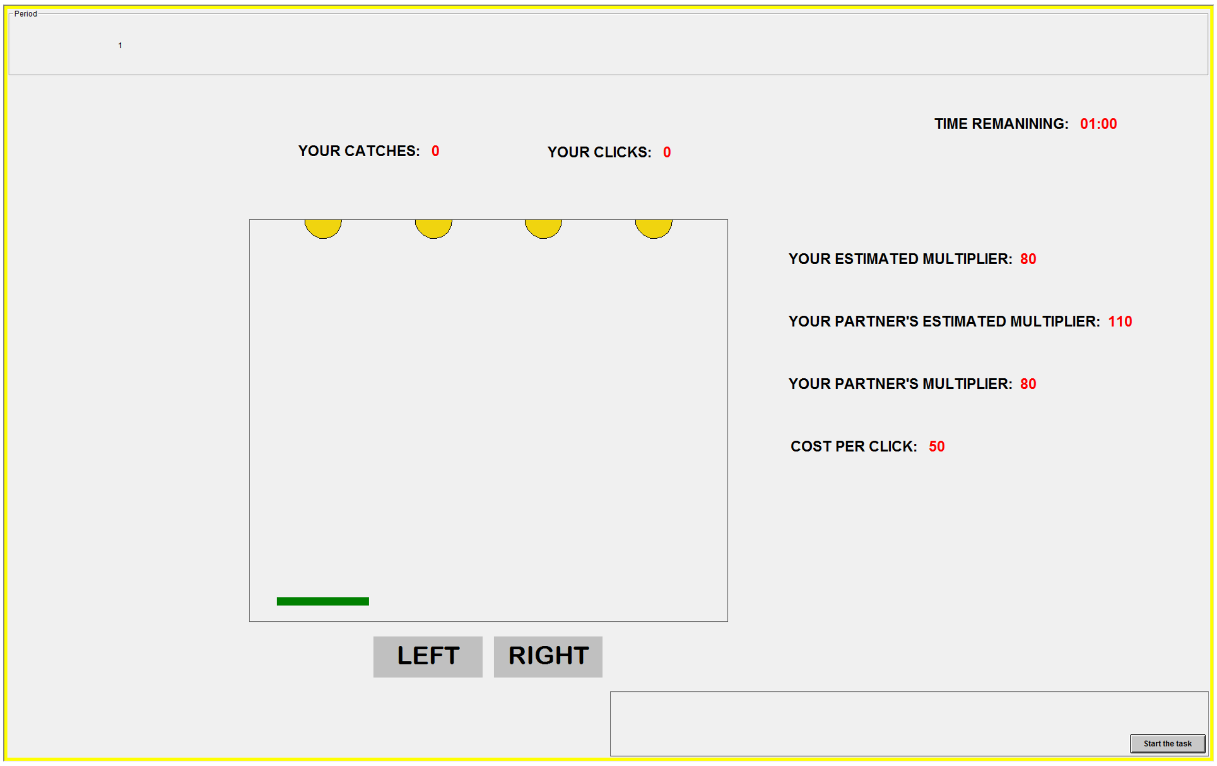

As you can see above, the task box displays the time left (in seconds), your number of clicks and catches (updated in real time), your estimated multiplier associated to your estimated rank in the quiz, your partner's estimated multiplier

associated with his/her estimated rank in the quiz, your partner's multiplier determined by his/her rank in the quiz, and the cost per click.

In **periods 9 to 16**, your multiplier, your catches, your partner's multiplier, and your partner's catches determine **your earnings** in tokens as follows:

your earnings = (common pot)/2 – your expense.

That is, in **periods 9 to 16** your earnings are equal to half of the "**common pot**" minus **your expense**. Your expense is equal to the cost per click of 50 tokens multiplied by your number of clicks for the period.

Similarly, in **periods 9 to 16**, your partner's earnings in tokens are determined as follows:

your partner's earnings = (common pot)/2 – your partner's expense.

That is, in **periods 9 to 16**, your partner's earnings are equal to half of the "**common pot**" minus **your partner's expense**. Your partner's expense is equal the cost per click of 50 tokens multiplied by your partner's number of clicks for the period.

In **periods 9 to 16**, the **common pot** is:

common pot = (your multiplier x your catches)
+ (your partner's multiplier x your partner's catches)
+ (5 x your catches x your partner's catches).

That is, in **periods 9 to 16**, the **common pot** is the sum of three components: **your multiplier times your catches** (**first component**), plus **your partner's multiplier times your partner's catches** (**second component**), plus **5 times your catches times your partner's catches** (**third component**).

At the end of each period from 9 to 16, you will see on your computer screen how many balls you caught, your clicks, and your earnings for that particular period.

Please note that catching more balls by moving the tray more often does not necessarily lead to higher earnings in this part of the experiment. This is because both **your pot** or **common pot** and **your expense** matter for **your earnings**.

Recall that your earnings in tokens will be converted into Swiss Francs at the rate of 300 tokens to CHF 1. The example that follows illustrates how your earnings are computed in **periods 9 to 16**.

Example: Suppose that, amongst periods 9 to 16, the one that counts for payment is 11. Imagine your multiplier is 120, you caught 20 balls, and you clicked 15 times. Imagine also your partner's multiplier is 160, she caught 25 balls, and she clicked 30 times. Then, we have:

first component of the common pot = 120 x 20 = 2400

second component of the common pot = 160 x 25 = 4000

third component of the common pot = 5 x 20 x 25 = 2500

common pot = 2400 + 4000 + 2500 = 8900

your expense = (50 x 15) = 750

your earnings = 8900/2 – 750 = 4450 - 750 = 3700

your partner's earnings = 8900/2 – (50 x 30) = 4450 - 1500 = 2950

Your earnings at the ball-catching task will be determined by only one period from 1 to 16. The period that counts to determine your earnings will be randomly generated by the computer and each period is equally likely to be selected.

This is the end of the instructions of this part of the experiment. Do you have any questions? If you have questions, please raise your hand.

Fourth Part:

Option to make another estimate

In this part of the experiment, we will ask you if you would like to make another estimate of your rank in the quiz. If your answer is "**no**," then the first estimate is the one that counts for payment. If your answer is "**yes**," then you have to provide a **second estimate** of your rank in the quiz.

The payment for your second estimate is determined in the same way as was the payment for the first estimate. That is, the more your second estimate of your rank is precise, the more likely it is that you will earn **600 tokens**. The precise way your earnings are determined is the same as the one described in the instructions of the second part of the experiment.

If you provide a second estimate, then the estimate that counts for payment will be randomly generated by the computer and each of the two estimates (the first one and the second) is equally likely to be selected.

This is the end of the instructions of this part of the experiment. Do you have any questions? If you have questions, please raise your hand.

**Fifth Part:**<br>Make distributional decisions

In this part of the experiment, you will make 10 decisions that concern you and another participant from your group, excluding your partner at the ball-catching task. The other person will be randomly paired with you. You will never learn who this person is, and the other person will also not learn of your identity.

In each of the 10 decisions situations, you have exactly two options, an option LEFT and an option RIGHT. Each option involves a monetary amount for the "Decider" and a monetary amount for the "Receiver." The 10 decisions situations will be presented successively on two computer screens with 5 decision situations in each.

The 5 decisions situations on the first computer screen will be:

**Dec. Nr.**	**LEFT**		**Your Choice**	**RIGHT**	
	**Decider earns**	**Receiver Earns**		**Decider earns**	**Receiver earns**
1	**360 tokens**	**585 tokens**	LEFT ○ ○RIGHT	**450 tokens**	**450 tokens**
2	**405 tokens**	**585 tokens**	LEFT ○ ○RIGHT	**450 tokens**	**450 tokens**
3	**450 tokens**	**585 tokens**	LEFT ○ ○RIGHT	**450 tokens**	**450 tokens**
4	**495tokens**	**585 tokens**	LEFT ○ ○RIGHT	**450 tokens**	**450 tokens**
5	**540 tokens**	**585tokens**	LEFT ○ ○RIGHT	**450 tokens**	**450 tokens**

The first column displays the number of the decision situation. The second column, the payments to Decider and Receiver when the Decider chooses LEFT. The third column is where you make your choice. You click in option “LEFT” if you wish the Decider and the Receiver to receive the payments associated with LEFT. You click in option “RIGHT” if you wish the Decider and the Receiver to receive the payments associated with RIGHT The fourth column displays the payments to Decider and Receiver when the Decider chooses RIGHT.

The 5 decisions situations on the second computer screen will be:

**Dec. Nr.**	**LEFT**		**Your Choice**	**RIGHT**	
	**Decider earns**	**Receiver earns**		**Decider earns**	**Receiver earns**
6	**360 tokens**	**315 tokens**	LEFT ○ ○RIGHT	**450 tokens**	**450 tokens**
7	**405 tokens**	**315 tokens**	LEFT ○ ○RIGHT	**450 tokens**	**450 tokens**
8	**450 tokens**	**315 tokens**	LEFT ○ ○RIGHT	**450 tokens**	**450 tokens**
9	**495 tokens**	**315tokens**	LEFT ○ ○RIGHT	**450 tokens**	**450 tokens**
10	**540 tokens**	**315 tokens**	LEFT ○ ○RIGHT	**450 tokens**	**450 tokens**

When the experiment is over, the computer will randomly choose one of the 10 decision situations to determine the payments for this part. The computer will also randomly choose whether you are the Decider or the Receiver. That is, the computer will randomly choose if the option you have chosen in that decision situation is implemented, so that you will be the Decider or, on the other hand,

if the option your matched participant has chosen in that particular decision situation is implemented, so that you will be the Receiver. It is equally likely the computer assigns you to the role of Decider or of Receiver.

In the case the computer assigns your option to be implemented you will receive the number of tokens corresponding to Decider in the chosen decision situation and your matched participant will receive the number of tokens corresponding to Receiver in that same decision situation.

For example, if the chosen decision situation was the 10th, the computer determined that your option is the one to be implemented, and you had chosen "LEFT", you would obtain 540 tokens while your matched participant would obtain 315 tokens.

If, on the other hand, if the chosen decision situation was the 10th, the computer determined that the option chosen by your matched participant is the one to be implemented, and your matched participant had chosen "RIGHT", then you would obtain 450 tokens while your matched participant would obtain 450 tokens.

Notice that the numbers in the example are just for illustrative purposes. They DO NOT intend to suggest how anyone may choose among the different options.

Please notice that, once all participants have made their choices, chance alone determines whether your role will be Decider or Receiver. Thus, the option you choose will only be considered if chance finally determines that for a decision situation it is your option the one being implemented. In case in the chosen decision situation your choice is not the one being implemented; your choice is simply not considered.

Therefore, in case your choice is not being implemented, your choice can affect in no way neither your payment nor the payments of any other participant.

At no time any participant will know the option chosen by their matched participant.

This is the end of the instructions of this part of the experiment. Do you have any questions? If you have questions, please raise your hand.

Sixth Part:

Make one risky decision

On the screen of your computer, you will see a square in which you can find 100 boxes.

You will earn 5 tokens for each box you will decide to collect. The collection process of boxes is automatic: each second, a collected box changes its color. The collected boxes change color beginning from the left top of the screen and updated accordingly.

Behind one of those 100 boxes is hidden “a bomb” that may destroy all the boxes that have been collected.

The “bomb” can be in either box with equal probability (the probability that a “bomb” is in a particular box is equal to 1/100). **Nevertheless, you do not know behind which box the “bomb” is located.**

Your task for this part is to choose when to stop the collection process of boxes. You can do it by clicking on the button “STOP” whenever you wish.

If you collect the box containing the “bomb,” the “bomb” will “explode” and you will not earn any tokens. If you stop the collection process of boxes before collecting the box containing the “bomb,” the “bomb” will not explode and you will earn the tokens that have been accumulated so far.

Notice that you will know whether one of the boxes you collected contains the “bomb” only at the very end of the task. If you collect the box containing the “bomb,” the “bomb” will explode only at the end of the task: this means that you may collect the box containing the “bomb” without knowing it.

We will begin this step by a training period. The goal of this training period is to show how this task works. Once the training period is finished, the task begins. The training period is only an example: you will not earn any tokens from the training period.

This is the end of the instructions of this part of the experiment. Do you have any questions? If you have questions, please raise your hand.

Seventh Part:

Complete a questionnaire

In this last part of the experiment, we ask you to complete a questionnaire. After completing your questionnaire, you will have to wait for everyone to complete theirs. After that you will be paid.

Your final payment is the addition of your earnings in each part of the experiment and of your show-up fee. We will print a page with your payments and hand it to you. You must take it with you outside the LABEX where a study administrator you use it to give you your final payment.

Please take note once again that 300 tokens in the experiment correspond to 1 CHF. If you have questions, please raise your hand.

Control questions: [not part of the experimental instructions]

Please answer the questions that will appear on your computer screen. The objective is to have complete clarity about the rules in the experiment. When you are done, click okay to proceed to the next screen. The computer will check if the control questions are answered correctly. If they are answered incorrectly you need to try again. Only after all participants in your group have answered these questions correctly can the experiment proceed.

1. Suppose you are ranked on the 7th position, what is your multiplier?

Answer: _______ [120]

2. Suppose you are ranked on the 11th position, what is your multiplier?

Answer: _______ [40]

Suppose that the period that counts for payment is period number 9. Suppose your multiplier is 160, you caught 40 balls, and you clicked 30 times. Suppose also your partner's multiplier is 80, she caught 30 balls, and she clicked 20 times.

3. What is the value of the **first component** of the common pot?

Answer: _______ tokens [6400=160*40]

4. What is the value of the **second component** of the common pot?

Answer: _______ tokens [2400=80*30]

5. What is the value of the **third component** of the common pot?

Answer: _______ tokens [6000=5*40*30]

6. What is the value of the **common pot**?

Answer: _______ tokens [14800=6400+2400+6000]

7. What is the value of half of the **common pot**?

Answer: _______ tokens [14800/2=7400]

8. What is the value of **your expense**?

Answer: _______ tokens [1500=50*30]

9. What is the value of **your earnings**?

Answer: _______ tokens [5900=7400-1500]

10. What is the value of **your partner's earnings**?

Answer: _______ tokens [6400=7400-50*20]

References

Gervais, S. and I. Goldstein (2007): "The positive effects of biased self-perceptions in Firms," *Review of Finance*, 11, 453–496.

List, J. A., A. M. Shaikh, and Y. Xu (2019): "Multiple hypothesis testing in experimental economics," *Experimental Economics*, 22, 773–793.

Romano, J. P. and M. Wolf (2005a): "Exact and approximate stepdown methods for multiple hypothesis testing," *Journal of the American Statistical Association*, 100, 94–108.

——— (2005b): "Stepwise multiple testing as formalized data snooping," *Econometrica*, 73, 1237–1282.

——— (2016): "Efficient computation of adjusted p-values for resampling-based stepdown multiple testing," *Statistics & Probability Letters*, 113, 38–40.

Westfall, P. H. and S. S. Young (1993): *Resampling-based multiple testing: Examples and methods for p-value adjustment*, John Wiley & Sons.
